# Manual of Human Physiology

**Published:** 1838-01

**Authors:** 


ART. VI.

1. *Handbuch der Physiologie des Menschen, für vorlesungen.* Von DR. JOHANNES MÜLLER, Professor der Anatomie und Physiologie an der Universität in Berlin, &c. &c. Erster Band. *Coblenz,* 1835.—Zweiten Bandes, 1ste Abtheilung. *Coblenz,* 1837.

*Manual of Human Physiology.* By DR. JOHANNES MÜLLER, Professor of Anatomy and Physiology in the University of Berlin, &c. &c. Vol. I. *Coblenz,* 1835. Royal 8vo. pp. 856.—Vol. II. 1st Part. *Coblenz,* 1837. Pp. 247.

2. *Elements of Physiology.* By J. MÜLLER, M.D. &c. *Translated from the German, with Notes,* by WILLIAM BALY, M.R.C.S. &c. *Illustrated with Steel Plates and Wood-Engravings.* Part I.—*London,* 1837. 8vo. pp. 428.

3. *Die Erscheinungen und Gesetze des lebenden menschlichen Körpers im gesunden und kranken Zustande.* Von DR. FR. ARNOLD und DR. J. W. ARNOLD, Professoren an der Hochschule in Zurich. Ersten Bandes, 1er Theil. *Zurich,* 1836. 8vo. pp. 388. — 2er Theil. *Zurich,* 1837. Pp. 460.—Zweiten Bandes, 1er Theil. *Zurich,* 1836. Pp. 253.—2er Theil. *Zurich,* 1837.

*The Phenomena and Laws of the living Human Body, in Health and in Disease.* By DR. FR. ARNOLD and DR. J. W. ARNOLD, Professors in the Academy at Zurich. 1st Vol. 1st Part. *Zurich,* 1836. 8vo. pp. 388.—2d Part. *Zurich,* 1837. Pp. 460.—2d Vol. 1st Part. *Zurich,* 1836. Pp. 253.—2d Part. *Zurich,* 1837.

4. *Rudiments of Physiology, in three Parts.* Part I. *On Organism.* Part II. *On Life as manifested in Irritation.* Part III. *On Life as manifested in Sensation and in Thought.* By JOHN FLETCHER, M.D., F.R.C.S.E., Lecturer on Physiology and on Medical Jurisprudence.—*Edinburgh,* 1835, 1836, 1837. 8vo. pp. 155, 146, 144.

5. *Outlines of Human Physiology.* By HERBERT MAYO, F.R.S. &c. *Fourth Edition.—London,* 1837. 8vo. pp. 534.

6. *Human Physiology.* By ROBLEY DUNGLISON, M.D., Professor of Materia Medica in the University of Maryland, &c. *Second Edition.—Philadelphia,* 1836. Two Vols. 8vo. pp. 546, 566.

I. THE author of the first of the works before us, though only in the meridian of life, has long achieved a celebrity which extends far beyond the limits of his own country; the just reward of his valuable contribution to every one of those portions of natural science upon which physiology is founded, and from which it derives the materials of further advancement and perfection. His earlier works are preserved principally in the national periodical publications; as the journals of Meckel, Tiedemann, and Ammon. They are papers of singular value and interest on subjects of minute anatomy in all the classes, with the physiological associations, and on the process of development. Whilst still at Bonn, he published, as an independent work, first, his Treatise on the Physiology of Sight, in which he takes up the subjective phenomena of vision, or (if it be now necessary to explain the term,) those changes in the living body which are intermediate between the refraction of light in the eye and the mental perception. Afterwards he gave to the world another work of surpassing excellence, his treatise *De penitiori structurâ glandularum.* In this great work,—from the injection of the ducts of glands, and of the vessels which supply these ducts,—from the mode in which the glands are first formed by continual subdivision, and, as it were, germination of the duct, (which begins like a cæcal production or tubular growth from the general mucous cavity, and shoots into the plastic matter of the germ; differently indeed in different glands, so as in some cases to form merely tubules with closed cords, in others tubules which expand into vesicles,) he proves beyond the possibility of doubt, with respect to all glands, that, however various the form of the subdivisions may be which the branching ducts assume, yet the object and purpose is the same in all. It is, by means of the convoluted and subdivided ducts, to secure a large surface in a small space for the disposition of capillary vessels. The internal surface of the duct, so disposed, is the secreting surface. The capillary vessels which supply that surface nowhere terminate in the ducts by open mouths. When most minute, the ducts are always still much larger than the vessels which Haller and others supposed were their beginnings; and the peculiar product of any gland is secreted from the internal surface of its ducts in a way exactly analogous to the corresponding function of any portion of the intestinal tube.

These various works, of which the results are now generally adopted in the publications of later systematic writers, have signalized their author as an accomplished anatomist, a practical microscopic observer, a good animal chemist, an honest experimenter, a learned commentator upon the entire literature of each subject he undertakes, a sound and philosophic reasoner.

Qualities so distinguished naturally pointed out the subject of them as destined for the highest situation, in which honours and his peculiar means of usefulness might be combined; and, according to the equitable practice of the Prussian government, whose decisions in the adjudication of medical distinction almost universally respond to the general voice of the scientific medical portion of its public, Müller now occupies at Berlin the chair of the celebrated Rudolphi. Honours, however, do not appear to damp his energies. We still find him unceasingly engaged in his labours; still distinguished by the same ardent zeal and inflexible perseverance; still producing either original works or continuing the works of his predecessors. His *Archiv. für Physiologie* is a continuation of Meckel's Journal; in which, not to mention his numerous original communications, he gives, at the end of each year, a comprehensive account and estimate of the value of the contributions to anatomy and physiology throughout Europe within the period: whilst, as larger original works, published separately, we may instance his anatomy of the Myxinoïdæ and the allied species, and his System of Physiology.

The last of these works, coming from a person so peculiarly accomplished for the task, has naturally excited a great expectation. We believe that, as far as it has proceeded, (for it is yet unfinished,) it has not disappointed those of the author's countrymen, who were disposed to be satisfied with the completion of what he undertook to perform. But it is not unreasonable to suppose that this work, if generally known in this country, would excite a different kind and degree of interest from that which has attended it in Germany; for there it is but one of many comprehensive productions which an ardent competition to advance the knowledge of vital processes has brought forth within a limited period; whilst the sobriety of philosophic speculation which its author evinces may scarcely, perhaps, be estimated at its just value by those who are familiar with the dazzle and the delight which poetry or mysticism in the garb of science may afford. Here, on the other hand, where two or three good compendiums of the science serve us for a period of twenty years, and these, however they illustrate the talent and the knowledge of their authors, from their design not full, and from the national taste not speculative,—here, where nevertheless there are countless numbers anxious to participate in the honours of advancing a science which formerly received so much from our countrymen, and still continues to receive not a little,—a work like Müller's would be received as a precious gift, comprising, as it does, all the important facts of the science; reducing them to their just value for the confirmation or the improvement of theory; holding prominently forward the lights which organic chemistry and comparative anatomy and physiology have afforded to the physiology of man; and clearly and constantly directing attention to the nature of those difficulties of processes yet unsolved, and to their bearings upon received opinions.

Since the preceding sentences, and indeed since the whole of the present article was written, Mr. Baly's version of a part of Müller's treatise has been published; and, after having compared a considerable portion of the translation with the original, we are bound to express our unqualified approbation of the manner in which Mr. Baly has executed his task. The translation is not only faithful and accurate, but elegant, and reads throughout (with very few exceptions,) like an original English work. We consider the English student, and indeed the whole profession, under great obligations to Mr. Baly for the very important addition which he has made to the medical literature of this country; and we recommend his work, in the strongest terms, to all our readers who do not possess the original treatise.

Although we regard Müller's work as by far the best of its class, still Germany is rich in productions of this general character, though with individual modifications. Such are the Physiology of Rudolphi, the delightful Biologie of G. R. Treviranus, the Physiology of Tiedemann, and that of Arnold. The first two of the four last named are broken off in the midst; the one by death, the other by the altered purpose of its author, who, finding that, in a long work interrupted by the vicissitudes of stormy times, the rapid growth of the science had rendered the early volumes old before the later could be brought forth, resolved to give the results of his labours in another form and in shorter commentaries. Of the other works, we trust the authors will be spared to complete their laudable undertakings. But, besides those already mentioned, many other systematic works on physiology have issued from the German press within a limited period: works of a loftier pretension and a much wilder flight, not always so well sustained as that more even course within sight of the world's realities to which we have alluded; yet still, in some instances, uniting the rare powers of high genius with the intelligence and the talent of patient research. We purpose, in the pages which follow, to take a short view of the course which physiological science has run in Germany since Haller's time; which may, in a few words, give some idea of its fortunes in the different schools of that country.

During the latter half of the last century, Haller's doctrines, founded on direct experiment, were sifted by his opponents and extended by his followers. The several modes in which the various organic tissues manifest their vital reactions were carefully investigated, and conclusions similar to those which Haller had established concerning two of these, the muscular and nervous, were extended to all. Each different organ and each different tissue was shown to have its own reactive energy, its own peculiar mode of life. Of these different energies the control and direction was assigned to a higher power in the living body, rather to satisfy, as it seems, the reason of man, which strives after unity of cause, than as the result of abstraction or higher generalization. This controlling power was differently assigned by different teachers. By some it was given to the nervous system; hence the theory of "nervous energy" as the cause of the peculiar "perceptivity" of the different tissues; whilst, according to Blumenbach, the various tissues and organs come into being with their several powers by the "Nisus formativus," or "Bildungstrieb." Of these two theories we may observe, that the first, instead of ascending in the chain of causation, assigns a partial effect of life, or, in higher creatures, a condition of its manifestations, as the proximate cause of life; whilst the other appears to assign a process for a cause.

Reil, who entertained the same idea of life,—viz. that of many manifestations conspiring to form unity,—revived, with great power of genius, the corpuscular theory of Descartes to explain its proximate cause. This cause is, according to him, the peculiar mixture and form of matter. The powers of organic matter depend upon elective attraction, upon its capacity to assume a special form in virtue of a crystallization which is not merely symmetrical, but (zweckmassig) appropriately adapted to an end. "The matter," says he, "which animal substances attract from without is attracted according to a determinate law, in which is contained the reason of its peculiar form. So the germ of a crystal of common salt attracts the component part which may be yet wanting to complete the perfect cube by a determinate law, in which lies the cause of that peculiar form." There are many passages in Reil's work which show him to have been no bigoted adherent to his own theory, to have been aware of its difficulties,—nay, of its imperfections. "We cannot define," says he, "what the power is which characterizes the matter of animals and plants, until chemistry is able to tell us more certainly what are the primary substances of which they are composed;"* and, as if uncertain of his own principle, he asks "are the phenomena of life the resultant of the sum of all the properties which are met with in organic matter? or, is there some single matter on which alone the phenomena depend?"† "To me it appears not improbable that, besides the matter which we perceive with our senses and can treat chemically, there may be yet some finer substance in animal bodies, of an unknown nature, which, being

* Reil's Archiv. für die Physiologie, vol. i. p. 48. † Ibid. p. 30.

commixed with that which is visible, may render it complete,—may, according to the measure and the mode of the addition, ennoble that which is coarse, and may render it capable of acting as the sufficient cause of animal phenomena, in all their determinate modifications." Thus does Reil elude the very principle of his system. In what, we may ask, does his "finer matter" differ from any imaginary Archæus? In explaining the facts of any one particular science, there seems no philosophy in determining to borrow from some other science, whose facts are not strictly analogous, the causes upon the assumption of which that other has been constructed. More especially is this indefensible when that other science, so far from being complete, is still obliged to have recourse to hypotheses to combine its phenomena. If that, which is a legitimate end and object of science, shall ever be realized,—the reduction of organic force to those general forces which animate and rule the universe,—it will then, as we believe, appear, not that ordinary physical or chemical powers characterize the class, but that limiting conditions have come into view, and are clearly recognized and accurately defined, and that, under the operation of these, the higher power is restricted to specific exhibitions of energy so very inferior. What these conditions may be has not yet been even guessed at.

But, though his theory was imperfect, as we have seen, yet the genius and talent evinced by Reil, in contributing to establish by reason and by induction the important truth that peculiar organic matter and peculiar vital properties are always in strict relation to each other, must rank him amongst those who have signally contributed to the advancement of physiological science. He constantly insists, as indeed was essential to his system, that organic power is only seen in connexion with organic matter. Irritability is not something infused into muscle, nor sensibility something superadded to nerve. In virtue of the power, whatever it may be, which brought these forms of matter into existence, these forms have, with their existence, their properties also. Matter and power are only known in mutual connexion; the existence of matter is action, its peculiar existence its peculiar mode of action. Every organ, therefore, bears within itself the cause of its own phenomena; by means of its own powers it is nourished, grows, performs its office in the animal economy; and, though its connexion with the rest of the body, as part of a whole, must necessarily be maintained, as a condition of the maintenance of its powers, yet this connexion is but a condition, and not a cause of those powers. "The animal body," says Reil,* "may be considered as a great republic consisting of many parts, each part indeed preserving a certain relation to all the others, and each contributing to the preservation of the whole; yet each part acts by its own peculiar powers, and possesses its own peculiar perfections." Muscular fibre is irritable because it is muscular fibre, not because it derives power from the nerves; though connexion with the nerves be one of the conditions of the maintenance of that power. In this same spirit, when speaking of the growth of bone, Müller expresses himself as follows:

"It is entirely erroneous to suppose that one organized part is the organ of nutrition for another organized part; as, for instance, that the substance of bone is

* Archiv. i. 105.

formed by the periosteum, the bone nourished by the periosteum. The substance of bone, inasmuch as it is organized, must itself assimilate. Unorganized parts alone, those which have no vessels, as hair, nails, teeth, the crystalline lens, are produced by an organized matrix and maintained by the apposition of new matter. I consider the opinion that bony matter is produced by the periosteal membrane as a piece of barbarism unworthy the present state of physiology." (*Phys.* i., 361.)

Many of the leaders of scientific research who succeeded Haller, were so closely engaged in establishing the nature and the varieties of vital power as exhibited in the different organs and tissues, that they seem to have paid but little attention comparatively to the circumstances which are indispensable conditions of its exhibition.* Men were recalled however from this exclusive view of the subject, by a theory (if it deserves the name) more partial and more exclusive than any which had yet been heard of,—but having another direction. Brown's "theory of excitement," to which we allude, was far more favorably received in Germany than elsewhere; for there it effected a signal revolution in medicine. Its adoption as a medical theory has been accounted for by the fact that on its first announcement the doctrines of Galen had not been entirely expelled from the country; and because a system remarkable, like that of Brown, for its simplicity and its slender requirements of labour or of learning, must needs recommend itself by contrast to many who were weary of the cumbrous apparatus of Galen, his humoral theory with its primary and secondary qualities. To the faults of Brown's system it is scarcely now worth while to allude: its occult quality "excitability" whose nature and laws are not even hinted at, its neglect of all distinction between the phenomena of life as apparent in the different systems, its reference of the blood to the class of mere stimuli, as if the animal fluids were not vital, its attributing the same value and effect to every stimulus, every one of them being assigned as a cause through whose efficacy the excitability, or vital power, is used up, whilst there is no cause assigned by which its losses may be repaired. These capital defects, with the theory itself, may be forgotten. The good which arose from its temporary adoption by numbers has been permanent. It recalled attention to the external conditions of life, to the effect of warmth, of air, of blood, of the secretions, of food, upon its manifestations. It was seen more clearly that unless such conditions be fitly ensured, however exact may be the form and composition of parts which organic power has brought into being, organic action is suppressed. "This inactive state," says Müller, "of the organic powers subsisting in the fecundated germ of the egg which has not been brooded, or in the seed of a plant before it has sprouted, must be distinguished from death. Yet it is not life, but specific capacity of life. Life itself, the manifestation of organic power, begins under the action of certain conditions,"† and can be maintained only whilst they are present.

The presence, then, of these necessary conditions of life is attended with those changes of the material composition of organized bodies, which are the essence of vital phenomena. The conditions themselves are, in

* This does not apply to Blumenbach, nor to Reil: they were amongst the first who recalled attention to the conditions of life.

† Phys. i., 28.

fact, the supply of new matter which combines with the various organs whilst some of their previous components are removed.

"The warmth, the air, the food, the animal juices necessary for the conversion of that food into blood, the blood itself, the imponderable matter which seems to be supplied to parts by nerves: all these things are the necessary materials for the production of those successive changes in organic matter which are the consequence and the continuation of life. Though some of these conditions be but ordinary forms of matter, binary combinations, yet their effects on the living organism are seen in very different productions. These are never binary combinations: such are only observable in substances which are undergoing separation, or have been separated, for removal. The carbonic acid, a binary compound, escapes from the blood in respiration; whilst the effect of the oxygen which in part unites with the blood is seen on its becoming engaged in those multiple combinations which are peculiar to that fluid."

"The vital stimuli must not be confounded with other stimuli which, though they have an effect upon the living body, do not add to its power, contribute nothing to the formation of organic matter. All that stimulates, all that produces change in the composition of living tissues is not therefore vital stimulus. A mechanical stimulus may modify the condition of a sensitive membrane, may call forth a manifestation of life, viz. sensation; yet it does not vivify, it gives no augmentation of organic force. But food is not merely a stimulus of organic tissues; it is itself capable of life: it is a stimulus which vitalizes and becomes itself vital. A healthy man may bear to be deprived of it scarcely more than a week: animals which stand high in the scale, not many weeks: amphibia, on the contrary, especially serpents and tortoises, much longer. Water, whether it enter into organic combinations, as water, or separated into its elements, is also required in its uncombined state for the manifestation of life: for animal parts which are not softened by water are not capable of life. Atmospheric air is so indispensably necessary for vital phenomena, that they cannot be maintained for many instants in the higher animals without respiration, without those changes of the blood connected with respiration, and without the influence of blood thus changed upon the organs. Privation of food may be borne for some considerable time: in amphibia, for instance, the blood may receive no new nutrient particles to present to the organs for a very long time. But those other changes which the blood brings about in the organs in virtue of the respiratory process cannot be dispensed with long even by them: by men only for a few seconds. Warmth also, especially important in the beginning of life, when the body is not in a condition to produce any, and in a general way indispensable for all organic things, whether plants or animals, would seem to enter into the composition of organic beings. For organic processes require a determinate temperature in every animal and every plant; and we know that in the binary combinations of ordinary chemistry, which cannot be effected without a certain temperature, a determinate quantity of heat is absorbed. Under the influence then of these conditions, food, water, air, warmth, organic being is spontaneously developed from its germ; the organic matter which is already present is constantly decomposed, and the vital phenomena themselves are phenomena of increasing combination of new matter and decomposition of old, or, in other words, of change in organic matter. Whether electricity be necessary for the development of life, is as yet quite undetermined." (*Müller*, p. 30-31.)

The degree in which life is dependent upon these different vital stimuli, comparatively, has been investigated by Spalanzani, by Schrœder van der Kolk, by Le Gallois, by Edwards, and others.

Thus have we seen that the investigation of the vital properties of tissue led the followers of Haller to conclude that there are in the same body different vital powers, and that they imagined some paramount force besides, capable of controlling them all. The examination of the conditions of life corrected what was illogical in this conclusion. For out of the examination, extended to all periods of the life of plants and animals, arose a knowledge of the process of development. This knowledge, though not perfect, is sufficiently complete to prove that the same essential power, which is connected with the matter of the germ at the time of its production by the parent plant or animal, evolves from each individual germ, by the conversion of matter which is present with the conditions of life, all those organs which belong to the purposes of its future existence. The germ is *potentially* that which the future body becomes *actually*. The vital energies of the organs are the development of the essential power of the germ.

We now turn to that which is more particularly understood as the German school of physiology, when views are alluded to which have been cherished to a greater extent, or at least more generally, in that country than in others. Germany has always, since she had a literature, been the nursing mother of philosophy; and philosophy has reacted there, as elsewhere, upon every branch of science. For what is philosophy in its true sense, the sense in which Bacon understood the word, but the science which endeavours to ascertain the principles of human knowledge, the scientia scientiarum? The system, therefore, from which these principles are sought must inevitably give the character and direction to the pursuit of every particular branch of science. The pursuit will be partial and the attainment unsatisfactory, until that first philosophy has established infallibly its own principles. Such systems being founded, all of them, in the nature of man's intellect, will be more purely speculative or more purely practical, according as the greater authority is attributed to his thinking or his active powers, his reason or his senses. But the true system must have both these characters, for the mind must exercise all its powers. Hence the great results in every science have been obtained by men who have laboured in this spirit. They are the genuine eclectics, who having gained their facts from experience, have attained to principles by meditation properly applied to facts, and have soberly and laboriously verified or corrected their principles by new practical applications of them. The earliest schools of philosophy in Greece, the Italic and the Ionic, took the different directions above alluded to. Pythagoras, the founder of the Italian school, sought in the reason of things, in the harmony of the universe, the cause of their existence. Thales, the founder of the Ionian school, sought it in the things themselves. The opinions of Pythagoras were expanded, by the noble conceptions of Plato, into the doctrines of the first academy; those of Thales by Aristotle, into the doctrines of the Péripatetic school. Plato asserted the doctrine of innate ideas, of universal notions, or forms of knowledge, impressed upon the mind of man by the supreme author of all things, of which the prototypes are in him, and appealed to them as the only source of all our knowledge of what is true, or good, or lovely; the fountain, from which must be drawn all certain principles of science, of morals, and of art. Aristotle descended from this lofty ground: he rejected innate ideas; the eternity of matter became a dogma of his school; and whilst all knowledge was declared to consist in the knowledge of causation alone, the appeal was made to the senses. To one or other of these classes of opinion, to that where reason or that where experience holds the highest place, may be traced most of the controversies which have since agitated philosophers. After the revival of letters, Descartes and Leibnitz supported the doctrine of innate ideas, which was rejected by Bacon and by Locke. But whilst Locke repudiated the doctrine of Descartes, he was far from enjoining such a reliance upon experience as admits nothing beyond sense. He did not deny that "the mind has some intellectual capacities, some powers accompanied with various ultimate laws of belief, for which no experience can account."* Locke's system was misinterpreted by Condillac, and was for a time received from him by his countrymen, in a form which led to the grossest materialism. According to Condillac, ideas are only "sensations transformed;" the faculty of feeling comprises all the other faculties of the mind. He sets up his human statue, allows it every mode of sensation, thus supplies it with notions of all external things; but forgets that reason and conscience are not thus imparted. The effect on physiological disquisition was soon felt. For, not to speak of De la Mettrie and the author of the Systême de la Nature, if such men as Cabanis could assert it to be "too obvious to require proof that physical sensibility is the source of all the ideas and of all the customs which constitute the moral definition of man," need we wonder at the revolting absurdity of St. Lambert, who proposed, as the formal definition of man, "an organized sensible mass that receives intelligence from all which surrounds it, and from its wants?" Leibnitz, on the other hand, modified the doctrine of innate ideas, so as, in part, to meet the objections of Locke; and after him the more ideal form of philosophy has passed through various stages of development in Germany, in the periods of Wolf, of Kant, of Fichte, of Schelling and his successors; and is still passing on, we trust, towards that adult and perfect form when it may be able to hold in its grasp, to embody and to vitalize all the results of experience and analysis, whether directed to matter or to mind.

The critical philosophy of Kant is an enquiry into the nature and the limits of the human faculties. Hume's scepticism was one cause of the enquiry, Berkeley's idealism another. If philosophy be a possible science, it must be able to reconcile such opposite results, and to shew in what they are opposed. What we perceive by our senses are not the things themselves; Kant endeavours to show that man is able à priori to deduce those laws of his reason in virtue of which nature stands related to him, and has for him a reality. He distinguishes between reason and understanding—the one transcendental, its conclusions necessary and general (like those of geometry); the other, judging by the senses, experimental, precarious. Ideas are the causal chain of the one, experience of the other. He applied his principles to organic bodies, gave his celebrated definition of an organism, and shewed that in each definite organism the final cause is the idea through which it is a part of the universal organism. The dark language of Kant's school was introduced into medicine and physical science by neophytes who seemed, with few exceptions, scarcely to know the sense in which the master had used it. The firm ground of experience and all the holds of practical knowledge appeared to be relinquished for the wild ocean of à priori dreams and distorted speculations.

Of the opinions of Fichte we need say little: he drew more deeply

* Dug. Stewart's Philosophical Essays.

than Kant had done the line of demarcation between the objects of perception and the perceiving mind, between object and subject. His principles have been criticised as being mere logical laws; forms of thought without substance; and therefore incapable of affording any knowledge of what either subject or object is in itself.

Schelling, as well as Fichte, proceeded from the critical school. His philosophy has had a general and a remarkable effect, having extended itself widely amongst jurists, philologists, physicians. His vivid imagination, his extensive acquaintance with nature, his great knowledge of the history and the philosophy of antiquity, enabled him, in appearance, to supply the deficiency which had been noted in Kant's system, to discover the uniting link, the common principle of its theoretical and its practical part. It is not the place here to enter into the particulars of Schelling's argument. He attempts the solution of the same problem, how it should come to pass that an external world exists for us: but far from separating subject and object, as Fichte had done, he shews that they are necessarily united. From consideration of the powers of nature and the powers of mind, he concludes that the laws of nature must be capable of proof immediately in consciousness as laws of consciousness, and conversely the laws of consciousness as laws of nature. Hence nature and consciousness are identified in the *absolute*, the infinite mind. The essence of the infinite is the absolute identity of subject and object. Schelling opposes absolute knowledge by means of *ideas*, (that intellectual intuition in which subject and object are one,) to the lower knowledge obtained by judgment through the senses. It seeks to know the essence and the form of things by ideas of reason, and considers being and knowing identical. It is transcendental idealism, and deduces all knowledge, not from nature and not from reason, but from that which identifies the two—the godhead,—and seeks to show the constant parallelism of mind and of nature.*

The chief propositions of the system, as applied to nature, are: that there is only one identical essence; that in respect of this there is only a quantitative difference; that the One Absolute reveals himself in the eternal production of finite things which are forms of his essence; that nature is not dead, but living, divine; that science follows this revelation as steps in the development of the absolute essence, and is an image of the universe whilst she unfolds the ideas of things.

It is no part of our business to show how arbitrary are some of the principles upon which this philosophy is founded, nor to point out its character of pantheism, of fatalism, of necessity. That has been done by others, and attempts have been made to correct these defects, whilst preserving its principle of identity. In fact, but for the marked influence which it has had upon physiology, and that a favorable one, we should not have alluded to it at all, but should have left it to the critics of cosmogonies in general, with those of Hesiod and Dante, and others. But since we hope to bring before our readers, as occasion may serve, several distinguished works by disciples of this school which presuppose a general knowledge of its tenets, and use its language, we have thought it convenient and right once for all to state what it is. When applied to nature

* Tennemann's Grundriss der Geschichte der Philosophie; p. 411, et seq.

in general, it is the theory of development in the largest sense: and, when properly understood, asserts the necessity of the closest observation of every part of the process, as preliminary to that intellectual intuition of the idea which connects the whole. As applied to living bodies, to particular living bodies, to particular organs, it is still the same. It enjoins the closest, the calmest, the clearest observance of process, all for the same end, to gain the ruling idea. The process is still found to be essentially the same: evolution of the absolute, conversion of the identical (in difference) into those opposites (forces) which are the visible qualities of things.*

Steffens applied to inorganic nature, and Doellinger and Oken to organic, the speculations which Schelling had himself chiefly illustrated by chemistry.

In connexion with this part of our subject we shall introduce a pleasing passage from one of a course of popular lectures on Psychology, delivered by Carus at Vienna. It is an exposition of the metamorphoses of plants: of the *idea* received by botanists from Goethe: and may serve two purposes; one to explain, by an instance, something of the language of Schelling's school; the other to shew in what light comparative anatomy, or morphology, is considered by it.

"Ordinarily, it is thought that we begin early enough if we trace the history of a plant from the seed. But the fact is here lost sight of that the seed is already a membered whole, and very far removed from that simplicity which gives the primordial phenomenon. That simplest form is to be sought in the first tenderest germ of the seed itself, within the flower of the parent, amongst the higher plants. Here in all respects the appearance announces itself as most simple; it presents itself in the most *indifferent* form, as globule; in the most *indifferent* consistence, as fluid; composed of the most *indifferent* element, as water. In a word, whether we contemplate a plant as the oak of a hundred years, or as the towering shadowy palm, or as a slender grass, or as the simplest thread of conferva, its first most simple form was a microscopic drop of fluid. This *unity* being given, as vital activity proceeds an internal membering of this drop arises, we observe how first the *opposition* between external and internal forms, husk and kernel, how in the kernel itself the opposition between mass that *affords* and mass that *receives nutriment* announces the future vegetable form; and so the seed, at length matured, is excluded from the parent body." . . . "Should now those circumstances occur under which its slumbering life must awake to new activity, then begins a new and very remarkable series of internal membering and parting. The germ from which the young plant is to proceed parts into plumule and root; the husks of the seed burst asunder, the nutrient mass heaped within forms and unfolds itself into the first compact root, or germ-leaves (cotyledons) of the young plant; and in the same exact proportion it shoots upwards joyously to the *light* by its green stem, and downwards into the *darkness* of the earth for nutrient juices by its root. But new divisions are still in progress: each new bud repeats in metaphor the seed, and the future metamorphoses are only distinguished from that original one in this, that the character of the latter becomes more delicate in them. Already the upper stalk-leaves assume a softer form, often variously divided, often, towards the flowering, discarding their simple green and dressed in various colours. In the calyx the form of the leaves is still further changed (sepals), they have a higher meaning, they enclose the flowers; until at length having reached their highest stage they unfold

* Even those who most resolutely resisted all connexion with the philosophical school, as Rudolphi and Tiedemann, conducted their labours in the spirit which it inculcated; witness their rich contributions to comparative anatomy, and the extensive research which they carry throughout animated nature when considering any physiological question. The age of rude empiricism has passed, as far as Germany is concerned.

into *opposites* in the petals and stamens. But this separation of petals and stamens in the flower is not enough: the relation of sex is indicated in the stamens themselves; and in the midst of opposites, thus carried to the highest degree yet still included in unity, comes forth the germ of a new seed. So that where one ring of vegetable life is completed a new ring begins, and links itself to the one chain which is extended through all vegetable life.

"From this example we learn the nature of an organic whole, its development from within outwards. We learn further from this, the 'genetic,' mode of considering nature, how many apparently different parts of plants are in fact only metamorphoses of one and the same fundamental form. And whilst we perceive that the transformations may be retarded or may be precipitated or may revert, the idea of unity in variety is but the more strongly infixed in us. We see, for instance, if it be the rule that leaves transform themselves into sepals, sepals into petals, petals into stamens: stamens may return again to petals, as in double flowers, as deviations from the rule: or petals to sepals, as on the points of the petals of the rose new sepals are frequently seen to shoot."*

Thus, however various may be the external appearance of a plant, the simple relation of stalk to leaf is intrinsically the whole plant. It can but metamorphose the same parts, and all the metamorphoses occur in succession in the same individual, being not transformations of organs but of the plant itself, viz. stalk and leaf. Hence there can be, as Müller has elsewhere observed,† no comparative anatomy of plants. The anatomy of one plant exhibits the transformations of the whole vegetable kingdom. In animals, on the contrary, the metamorphoses whether of body or of organs do not occur in the same individual. They are found in permanent forms scattered throughout the whole animal kingdom. Comparative anatomy, in determining these differences of form and of organs, does not rest satisfied with the result of its comparisons, which are so far only an aggregation of isolated facts. It endeavours to know the *process* of change; it endeavours, by looking through the animal kingdom, to gain the idea which directs the process, and so, intellectually, to be able to repeat it.

But to return from this digression. It may be said, and has been said, that the philosophy of nature commenced in fanciful dreams and arbitrary assumptions. And certain it is that it has failed to realize its magnificent anticipations. But the physiology which was connected with it did not always continue to soar in those lofty regions of unsubstantial speculation, from which it was directed to take its first flight. By an analysis of facts, it enquired into that mutual and intimate connexion of all things which had been asserted on à priori reasoning; the foundations of the science were thus largely extended. It investigated the relations which had been asserted to exist amongst living things; a higher zoology was the result, together with most valuable contributions to comparative anatomy. It fully tested the fundamental philosophical principle of unity in the midst of diversity; the history of development of the animal kingdom in general, of separate animals, of separate organs, was in consequence, so greatly enriched in substance and improved in form that a large share of the merit of its production appears to be due to the Germans. That was not merely a fanciful system to which such men as Doellinger, Meckel, Oken, Carus adhered. In their hands physiology

* Carus, Vorlesungen über Psychologie; p. 15.

† Vergleichende Physiologie des Gesichtsinnes; p. 28, 29.

assumed a determinate tendency, and its huge mass of facts became animated by a connecting idea.

Seeing that such have been the results obtained in Germany, we naturally ask—what is in fact the peculiarity of their system? and, if it be peculiar, in what respect it differs from those views which conducted Harvey, and Cuvier, and Bell, to their great discoveries? We do this the more willingly, because, though strongly averse to those crude hypotheses which are often advanced under the name of theory, we are not disposed to agree to the proposition so emphatically asserted by many; that physiology, being a physical science, is to be advanced by experiment and observation alone. It may safely be denied, we believe, that this is true of any physical science. Certainly it is not true of astronomy, for instance, the only perfect one of them all. For if it be asserted that this science is founded solely in the ascertained facts of mechanics and of the observation of the heavens, we may ask how it happened that the first law of motion was ever believed to be true, when it is found to be contradicted by every appeal that can be made to experience. We may ask, if observation be all in all, how it happened that Kepler, who knew the true motions of the planets, and announced those general laws respecting them which are, in fact, all that is necessary in order to determine the proximate cause of those motions, how he yet failed to assign a true cause? So far was he from the mark, that he believed the planets to be urged in their elliptic curves by forces which act in the direction of those curves. Yet Newton arrived at the true cause, and asserted its law by a series of inductions which were founded on Kepler's results alone. He arrived at it, however, by doing something which Kepler omitted; in German phrase, he discovered the "idea," the idea appropriate to those valuable facts which Kepler had ascertained after labours and disappointments innumerable, the idea which enabled him to produce them all in thought. He saw the principles included in the facts. *If* equal areas be described, according to Kepler's ascertained law, in equal times, then Newton saw that the body *must* be drawn towards the centre. *If* the orbit be an ellipse, then Newton saw that the force, drawing towards the focus, *must* diminish as the squares of the distances increase. *If* the squares of the periodic times of the planets be, as Kepler shewed, proportional to the cubes of their mean distances from the sun in the focus of all their elliptic orbits, then Newton saw that these bodies *must* gravitate towards the sun in the ratio of their masses directly, and the squares of their mean distances inversely. The assumptions are all matters of observation; and of these assumptions Newton's principle is the necessary result. But it required the mind of Newton to discover the ideas which suited the facts of the case, and to connect the two by evidence of the clearest reason.*

It is plain that, in this case, the most accurate observation of facts, the comparison of them in every possible relation did not of themselves lead to theory; to the comprehensive principle which, being once clearly seen, is capable of being expanded into all the details of the phenomena, and of confirming its own truth by shewing that phenomena which at first sight appeared subversive of the principle, are in fact its necessary con-

* La Place, Exposition du Systeme du Monde; p. 428.

sequences, "regular then most when most irregular they seem." The history of this science instructs us further that, even where general laws have been stated in their simplest terms, labours and difficulties different in kind but scarcely less formidable, yet remain: viz. the inductions of verification. With these difficulties astronomy has maintained a victorious struggle from Newton's time to the present. But what, let us ask, might be expected to be the result of such a struggle with respect to other sciences whose facts are more complicated? If in chemistry, if in physiology, the law of molecular motions apparently so anomalous were assigned, can we hope that we should be able to effect the useful and practical application of it, the deduction of all the particulars from the theory?

Whilst, therefore, it cannot be doubted that laws exist by whose operation organized bodies with their several members are evolved, for constancy implies law; yet, since they have not been discovered, equivalents or substitutes have been sought for them.

Such equivalents are found in observation of the results of these laws; or, of the processes which they are actually effecting; in observation directed to ascertain not the connexion of *cause* and *effect*, but of *means* and *end*.

"In considering the intellectual processes by which Harvey's discoveries were made," says Mr. Whewell in his admirable work just published, "it is impossible not to notice that the recognition of a creative purpose, which, as we have said, appears in all sound physiological reasonings, prevails eminently here. 'I remember,' says Boyle, 'that when I asked our famous Harvey what were the things that induced him to think of a circulation of the blood, he answered me, that when he took notice that the valves in the veins of so many parts of the body were so placed, that they gave a free passage to the blood towards the heart, but opposed the passage of the venal blood the contrary way; he was incited to imagine that so provident a cause as nature had not placed so many valves without design; and no design seemed more probable than that the blood should be sent through the arteries, and return through the veins, whose valves did not oppose its course that way.' We may notice, that this discovery implied the usual conditions, distinct general notions, careful observation of many facts, *and the mental act of bringing together these elements of truth.* Harvey must have possessed clear views of the motions and pressures of a fluid circulating in ramifying tubes, to enable him to see how the position of valves, the pulsation of the heart, the effects of ligatures, of bleeding, and of other circumstances, ought to manifest themselves in order to confirm his view. That he referred to a multiplied and varied experience for the evidence that it was confirmed, we have already said. Like all the best philosophers of his time, he insists rigidly upon the necessity of such experience. In every science, he says, be it what it will, a diligent observation is requisite, and sense itself must be frequently consulted. We must not rely upon other men's experience, but our own, without which no man is a proper disciple of any part of natural knowledge. And by publishing his experiments, he trusts, (he adds,) that he has enabled his reader to be an equitable umpire between Aristotle and Galen; or rather (he might have said,) to see how, in the promotion of science, sense and reason, observation and invention, have a mutual need of each other." (Vol. iii., p. 401-2.)

Again, in Cuvier's theory of the "conditions of existence," there is found such a substitute as that above alluded to. This theory we are glad to instance, because it embodies many of the views which have been considered peculiar to the philosophical school. Though the law of organic formation cannot be stated, yet it is found to be a result of the law not only that certain organs coexist, but that any modification of one of them necessitates corresponding modifications in all the others. "Animals with hoofs must be herbivorous, they have no means of seizing their prey. Having no other use for their anterior extremities than that of support, their shoulders require a less vigorous organization; hence the absence of the clavicle and of the acromion, and the narrowness of the scapula. Not requiring to perform rotation of the forearm, their radius will be united to the ulna, or, at least, articulated by ginglymus, not by arthrodia, to the humerus, &c." These views seem tolerably evident when once stated. Yet to embody them in a theory it required two things: one, to know the general character of all the groups and the details which modify their anatomy throughout the whole animal kingdom; the other, to gain the idea which subordinates these.* That idea is teleological: the organs are seen to have a purpose; it is seen that they conspire, in their several purposes, to effect a determinate useful result; it is seen that the various organs are associated for those ends by which the animal fulfils the purpose of its being. When this idea is verified in the details, it is found that there is *not merely a general but a specific consistence in the form and combination of organs; there is found to be a constant harmony between organs apparently the most remote; the altered form of one is invariably attended with a corresponding alteration in the others;* a fragment of bone, a process or a surface has a determinate character; an articular surface may indicate, to those who can read the enigma, the entire form and habits of the creature to which it belonged.†

By means like these connecting bands have been discovered between the limited intellect of man and the complicated results of vital forces, and which have proved their value almost as effectually as if, having assigned the highest physical cause, he was able to deduce those results as its necessary consequence. Neither, as has been seen, are the views suggested by the consideration of final causes, and those peculiar to the philosophical school of physiology opposed to each other like the poles of a magnet. On the contrary, they run parallel. But the ken of the former does not reach so far, neither does it penetrate so deeply beneath the surface. The teleological school, (say the philosophers,) is satisfied with partial views, with a certain seeming fitness on the surface of things; with evidence of artist-like design, with the belief that one thing is made for another, or for its own preservation. There is, however, (they add,) not merely this external fitness, but also an inward essential connexion of all things, pervading all and uniting all, both internally and externally. Nature is a system of connected parts; an admirable unity of innumerable members; the collection of these forms the organism which comprises them all. Man himself is such a member; an isolated member he is apt to think himself; but, in fact, a member whose existence is bound up with that of all the others in a totality in which all are included.‡

We now leave these considerations: in fact, as far as Müller's work is concerned, we are not required to recur to them. For it is practical: it is conducted in the true spirit of "thinking experience."

"I have made it, in all instances, my object to investigate the difficulties of the

* Whewell's History of Inductive Science; vol. iii., 476. Compare.

† Cuvier; Ossements Fossiles; vol. i., 49.

‡ These statements are collected from Valentin's new work, "Handbuch der Entwickelungs Geschichte der Menschen."

subject, and to analyze the more important facts in such a way as to lead to the solution of physiological problems; even the more abstruse, if our general knowledge of the principle of life were more complete. We deceive not ourselves with general formulæ of life, and so forth. Still the exact natural philosopher, who does not employ the term 'life' for every species of activity, is not so blind as not to perceive that every where in nature there is action. This every one knows. Activity is everywhere: even the rest of material particles is effected by their mutual attractions. Had custom originally applied the term 'life' to the activity of the system of the universe, I should have used it in this sense; but custom has limited it to the activity of organic beings. He who chooses to call the heavenly bodies 'organisms' may do so; in my youth I thought it quite right to do so. The consideration, however, that the differences of these extraordinary and ordinary organisms are greater than their resemblances, determined me to drop a designation so agreeable to some enquirers into nature." (*Müller*, Pref. to Part II. Vol. I.)

II. The second work announced at the head of this article, comprising the Physiology of Man in health and in disease, is executed by two brothers, professors at Zurich; the Physiology of Health by Dr. F. Arnold, well known for many works, particularly by his excellent treatise "Der Kopftheil des Vegetativen Nerven-Systems," which he published when a teacher at Heidelberg; the Physiology of Disease, by Dr. J. W. Arnold. It is with the former portion only of this work that we are at present concerned. The author premises a view of general Physiology, as introductory to the exposition of the special vital processes. In this he expounds not only the general phenomena and laws of the living body, but also its relations to external nature, and those conditions of its existence which are founded in its organization. Such a prefatory work he considers to be indispensable, if an author would avoid the inconveniences which appear inseparable from the plan of those who postpone the general physiology to the special; such, for instance, as the necessity of continually assuming as known that which is afterwards to be taught, or of inverting the nature, order, and connexion of vital processes; as is the case when the doctrine of the formation and motion of the blood precedes that of the chyle, when the doctrine of digestion follows that of nutrition and secretion, &c. In the general physiology the fluids, the tissues, the systems of the body, their properties and composition, are considered by our author, as far as this is necessary for the full comprehension of the special processes of life. He, moreover, in this part of his work, compares the organization, the corporeal and the mental faculties of man and animals, and of different races of men, since no aspect of human nature ought to be neglected by the physiologist. To this mode of treating the subject, it may perhaps be objected, that it forces into physiology many subjects which belong more properly to the province of natural history on the one hand, and of minute anatomy on the other. They who thus object will probably prefer the more concise and very elegant prolegomena of Müller, as sufficiently preparatory for a well educated student of medicine, whilst the special details are not so extensively anticipated.

Arnold, with the greater part of modern physiologists, considers life as the "aggregate of the phenomena presented by those natural bodies which have within themselves, under a certain form of being, the proximate cause of those phenomena; and which indicate, in all their processes, self-subsistence and fitness for an end." "These bodies," he says, "are called *living*, or *organic*, which are not merely subject to the ordinary laws of nature, but in which a special force operates, which in this respect is called *vital force*. Dead, or inorganic bodies, on the other hand, are all those whose manifestations and changes may be referred to the ordinary natural agents, called physical forces." "The form in which the life of an individual manifests itself is called 'organism:' its peculiar constitution 'organization.' The organism is a whole, consisting of parts necessarily and intimately bound together, and acting towards an end, viz. for the purpose of its own perfection and conservation." Again, "organic bodies exist not merely by and through themselves, but their life also depends upon a constant mutual action between them and the external world. For from it they incessantly receive matters, and to it they return matters, and stand in a reciprocal relation to the various forces of the universe which is indispensable to their continuance." (*Introduction*, § 2, 3, 4.)

The author's general enunciation of his opinions respecting the nature and properties of living bodies we present to our readers in full. They are characteristic of the school whence they originate, and illustrate some of our preceding remarks.

"Those phenomena presented by organic bodies, in the reception, the assimilation, and the rejection of matters, in respiration, in the motion of fluids, in nutrition, inasmuch as they possess the common character of being manifested by motions, justify the assumption of a single cause, or a single force capable of producing in the different parts the same, or at least corresponding phenomena. But these essentially differ from the effects of chemical or mechanical forces; nay, are manifested in opposition to them. The force, then, which is assumed as their cause, must be peculiar. It may be designated from the phenomena, the motor force (vis motoria); or, since it is apparent in all organized beings, organic force (vis organica); or, since it is the source of the chief processes in plants, vegetative force (vis vegetativa.) According as its manifestations are accompanied by change in space *without* contraction or *with* contractions, it has been considered under two different appellations, viz. *plastic force* and *contractile force*. And these again according to the variety of their effects have received different names and been considered as special forces. The first of them as *assimilative, absorbent, secreting, nutrient* force; the other as *muscular* and *contractile* force. Besides the phenomena due to these various forces, which have their common character in one and the same cause, there are other phenomena manifested in psychical activities. These appear later than the corporeal: they are peculiar to animals. In the lower orders of being they appear as sensations and desires, are gradually developed into perceptions and resolves; on these are founded the phenomena of thought and the accomplishment of purpose. Next come the processes which are accompanied by fancy and memory.

"All the phenomena of psychical life, (like those of corporeal,) admit of reference to one force, as the cause of the processes of the understanding and the will; and which many (Descartes, van Helmont, Stahl, Whytt, Bianchi, &c.) have designated as the one fundamental force of animal life. It is ordinarily called, from the system through which it operates, *nervous force*; but, from its essence, it may be more conveniently denominated *psychical force*." . . . "The aggregate of manifestations and effects of organic force is called *body*; the complex totality of the phenomena of nervous or psychical force, *soul*. The two are necessarily and intimately bound together in man, and cannot be separated even in thought; for they mutually determine and cause each other. They are powerfully and multifariously interwoven. Psychical life is related not merely to the understanding and the will, but to the corporeal processes also. And these in turn, but more especially the total amount of formative activity, have an effective reaction upon it. Body and soul together make up a whole which is exhibited to us in its highest perfection in the human organism.

"Since corporeal and psychical life are, in man and animals, thus essentially united and mutually dependent, a remote cause of life is assumed, which calls forth the organism in form, and originates its activities—the *vital force,* (vis vitalis of Blumenbach, Barthez, Sprengel.) This force it is which produces from fluids the primitive organic form, the globule or vesicle, and causes these phenomena of inherent activity which display themselves in the elementary form as effects of an internal cause and of fitness for an end, (Zweckmassigkeit.)"

"Since man exists in a world which supplies the materials of that constant interchange by which he repairs the powers which have been exhausted in vital processes, he must possess, in his various parts, the property of being susceptible of external influences, that he may act in consequence of the affection received from them. This susceptibility (facultas percipiendi,) exists in every part; and, inasmuch as it is a common property, corresponds to the vital force, of which it is a necessary predicate, being displayed in various degrees in the various structures of the body. External influences which determine the living body to action are called *stimuli.* The property of the organism, in virtue of which it is capable of reaction or of vital manifestations on the application of stimuli, is called *excitability,* (incitabilitas.) The condition of the body, and of its parts, thus induced, is called *excitement,* (incitatio, reactio.)

"Susceptibility (perceptivitas,) is present wherever there is vital force. As that force is displayed in two different directions, body and mind, so also are there two directions of susceptibility. As a property of an organism by which it perceives stimuli which determine it to reactions, consisting in changes in space, it is present wherever there is organic force. It is then called *irritability.* As a property of the human body by which it perceives external impressions, through which sensations are produced in the organs and structures of the body, it is confined to animal organisms, is a predicate of psychical force, and is called *sensibility.*" (§ 301 et seq.)

In those which Arnold gives as the general results of observation and reflection directed to vital processes, we find too much of the language of ordinary physics; and too little of accuracy in the proof that all the various so-called "forces" are but specific expressions of one and the same fundamental force. There is, in short, the same defect here as that which we alluded to in the system of Bordeu, Barthez, and others. Besides this, to speak of "a force of secretion," "of absorption," &c. may lead the inexperienced to suppose that these are real entities; that they explain the facts which are referred to their agency, without reference to the structures in which the phenomena occur. But this cannot be the intention of Arnold; and, if not, the term "property" might in all cases, except that of the highest cause, be substituted more conveniently for that of "force." But even this exception leads us again to the point from which the opinions of physiologists diverge so widely, according to the philosophical notions which they may have imbibed. With one set, force and matter coincide, force is a property of matter; with another set, matter is either a production of force, or force is something superadded to matter. They are conceived of as distinct things: force as an entity which does not occupy space; vital force as the architect and preserver of the body.

III. This question leads us to notice the third of the works which we have placed at the head of this article,—that of Dr. Fletcher, which he modestly terms "Rudiments of Physiology." It is the work of one who was enriched with much learning, and practised in original thought upon the philosophy as well as the phenomena and laws of life. The third part, at least as far as the composition goes, may be considered as the work of Dr. LEWINS, who has very lately given it to the world from notes of his deceased friend. We are led to notice this work in connexion with German physiology, because its author was well read in the works of German physiologists, and ably defends many of their tenets; and because we have thought that, to point out this character in a work of so much general talent, might be acceptable to many of our readers. We cannot pass over Dr. Fletcher's excellent chapter "on the Aggregation of Organized Beings," so rich in anatomical details, so cautious in generalizations, so just, in most instances, in its criticisms upon several subjects of much interest and difficulty,—without pausing to regret that one so admirably adapted to the office of a teacher of physiology should have been removed by an early death from his high undertaking.

With respect to the fundamental question to which we have alluded, Dr. Fletcher is altogether opposed to the admission of a "vital principle," in the sense in which it is understood in the works of Harvey, Stahl, Hunter, Whytt, Sauvages, Bordeu, Bell, Prout, Kirby, and others,—viz. an elementary substance, which, entering into certain aggregations of matter, becomes the cause, not only of the peculiar form of bodies, but of their physical properties and of all their phenomena. This notion, derived from the earliest times, recommended itself to men's minds when they observed the mysterious change which all living bodies undergo in death; a change so remarkable, that ordinary language ascribes it still to the extinction of the "vital spark," the abstraction of some finer spirit, or "breath of life;" and still more was it recommended by their persuasion that they knew the essence and nature of those grosser forms of matter which surrounded them, and from which living bodies appeared so greatly to differ. A stone was with them essentially "inert;" if it moved, some external force must act upon it: bodies, therefore, which moved spontaneously must possess within them some cause of their motions distinct from their matter. In later times, says Dr. Fletcher, it is not by such reasoning that a substantial vital principle can be defended; for it is known that properties essentially different result from differences in the composition, aggregation, and substance of different forms of matter.

"If we reflect on vital actions, anomalous as they appear, what do we find in them, in fact, but certain movements of either particles or masses of matter, not certainly identical with, but still very analogous to those which, in unorganized matter, we call chemical and mechanical; and which we are contented to ascribe, not to any substantial principle of action, but to certain properties and powers resident in these matters, the reciprocal action of which gives rise to what are called attraction and repulsion? And why need we hesitate to admit that similar, though not the same, properties and powers may, in organized beings, be competent, while they are in mutual co-operation, to effect those actions in which life consists, and which, of course, terminate on the cessation of this co-operation, as the ingredients of a chemical compound cease to be agitated when their affinities are satisfied, and a watch stops when either the susceptibility of motion in its wheels is destroyed, or the requisite power ceases to operate upon them? It is true a living body appears to require no such additions of new ingredients as a chemical compound, and no such frequent winding up as a watch, to avoid falling into the soon-established repose of the particles or masses of inorganized matter. But we must keep in mind that, in the latter case, while the properties and powers of the substances in co-operation are soon satisfied and exhausted, there is no inherent renewal of these substances, and with them of those properties and powers to renew their proper actions; whereas, in the former, it is the specific end of some of these actions to give rise continually to new aggregations of matter, distinguished by the same properties, and acted on by the same powers, as the old which have disappeared, so that the conditions of continual action are never for an instant suspended. It is not, then, that there is in living beings no addition of new ingredients, and no winding up, but that this addition and this winding up are incessant: and all that death implies, therefore, is a cessation of these as a necessary condition of life, in its character of a substantial vital principle on which such action depended.

"But is not, it may be asked, design, such as can be attributed only to some such vital principle within them, manifested in the heaving of the chest, the contractions of the heart, and the other sensible motions of various parts, as subservient to the several functions of organized beings; and still more in the various molecular actions, whereby, with undeviating accuracy, every particle of the machine is removed and deposited precisely at the time and in the quantity that is required, not only to form these several parts,—the eye or ear, for example, those finished examples of exquisitely adapted workmanship,—but subsequently to maintain each in a state of integrity, ever varying, yet ever the same? Undoubtedly; design deep and wonderful; . . . but this design is that of the great First Cause of all things, who has adapted in every case the physical causes, the immediate means, to the end to be fulfilled: not that of this miserable means, which acts and can act only in blind obedience to the laws imposed upon it." (Part II. *a.* p. 26, 28.)

After enumerating various instances in the inorganic kingdom which seem to him to be no less obvious proofs of the operation of final causes than those witnessed in organized matter, Dr. Fletcher proceeds:

"There is nothing, then, in reality more singular in the actions of organized beings than in those of unorganized matters,—nothing more indicative of design,—nothing more obscure,—nothing which stands more in need of a substantial principle, or resident entity, to account for it. We may, indeed, if we please, in conformity with the views of many philosophers, not only ancient but modern, call these actions of inorganized matters, chemical and mechanical, their life; but then, to be consistent, we must either allow, as the ancients did, a vital principle to inorganized matters, in common with organized beings, or we must refuse to the latter a principle of which the former are almost universally regarded in the present day as destitute. It is not contended that we understand the nature of any of these actions; it is not contended that we advance one step towards explaining them by ascribing them to the agents in question. But it is contended that we know the nature of vital as well as of either chemical or mechanical action; and that, if we are satisfied to attribute the two latter to the agents above specified, as is almost universally done, we cannot, without great inconsistency, refuse to ascribe the former, which are in every respect so analogous to them, to similar agents, instead of to an imaginary substantial principle, of the operation of which there is no more proof or probability in the one case than in the other. Of the immediate nature of physical causes in general we know absolutely nothing, since we are capable of recognizing their existence only by their effects. . . In like manner, we know not how or why a certain aggregation of matter called organized should be capable, when acted on by certain appropriate powers, of manifesting the phenomena of life. But we know that it does so; that, the more perfect is the organism, the more remarkable are these phenomena, and that any change in the former produces a corresponding change in the latter: and what other proof can we require or possess that organized matter is, *qua* organized, irritable or endowed with vitality, and that it is not upon any substantial principle of life that these phenomena depend?" (Ib. *a.* p. 29, 30.)

Thus irritation, or life in its simplest form, according to Dr. Fletcher, consists in the perception, by organized beings, of certain stimuli acting upon them, otherwise than either strictly mechanically, or strictly chemically. Sensation, also, according to him, is a perception also; but it differs not only in degree, but also in kind. The being manifesting sensation, not only perceives, but perceives that it is perceiving; in conformity with the view of Glisson. In the case of irritation, the susceptibility is called irritability, and the power which calls it into action may be of various kinds. In the case of sensation, on the contrary, the susceptibility consists in a new property called sensibility, and the power by which it is called into action is always irritation. By this is understood that, when an agent acts upon a sensible part, it does not immediately act upon the sensibility of that part, and thus excite sensation; but upon its irritability, exciting in the first instance irritation, which irritation becomes now a new power, and operates upon sensibility so as to produce sensation.

We purposely avoid entering upon the all-important question of the *immortality* of any portion of man's nature, however designated, as not necessarily connected with the question of life regarded as the subject of physiological enquiry. It is, however, but justice to Dr. Fletcher to state that he is a very strong advocate for the immortality of the *soul*, which he considers as something quite distinct from *mind*.

It is amusing to see how extremes meet. One party of physiologists, the various classes of Stahl's followers, assert that life and soul are but different names for the same essence; an essence superadded to body, which evinces itself as formative power in building up and sustaining the body, and as psychical power in those animals in which the brain is developed; whilst, in the highest of these, this psychical power displays itself as self-consciousness,—i. e. without any intermedium of matter. To this party they who hold Dr. Fletcher's opinions are, in appearance, altogether opposed. They justly say, that we know nothing of essences as distinct from matter; that force is only a name for the supposed cause of phenomena which affect our senses; that for every phenomenon there must be a material substratum, otherwise it could not affect our senses. But they overlook the fact that we have consciousness of much that cannot, according to this interpretation, be called a phenomenon at all, since it affects no external sense,—viz. the operation of our own minds. According to the Stahlians, the soul acts upon matter in every part of the body, and in the brain stands forth as the highest flower of animality, in its own unmixed character, resting upon body on the one side, and free from it on the other. With the second party mind is in body also, but only as the complicated function of its highest structure, the brain. According to the apparent admission of both, mind does act upon matter, and matter does act upon mind; whilst they can never agree to what extent this mutual action may occur, nor in what we should understand by matter, and what by mind. Dr. Fletcher asserts, as we have seen, the existence of a higher principle than mind, the immortal principle; but with what part of the body it is connected is not very apparent. It can scarcely, in his system, have any locality; for, if it have, it must appear as an organ or as a faculty, and in either case be transitory and perishable. Until this new principle is introduced, it is not quite clear that there is any great difference between Dr. Fletcher and Stahl; nor does there seem any necessity to superadd to the mind of man this distinct principle in order to secure to him an eternal existence, which is supposed to be denied to brutes. The Creator of souls may give to some that imperishable nature which he has denied to others.

But we are here touching, with Dr. Fletcher, upon subjects quite beyond our enquiry. Even in objects of legitimate physiological research, we are soon taught how incapable we are of comprising many of those infinite relations of which its ordinary phenomena are the results,—as secretion, nutrition, &c. Yet these remarkable processes are effected by secondary means, the Creator working by laws impressed upon matter; and it is still the same in the yet more astonishing phenomena of sensation and of thought. And, if the process still be the same in the creation of such intelligence as the human soul, we can have no conception, the most distant, of that infinite wisdom which is able to impose laws whose operations are necessary, and which yet leave the will free and the conscience responsible.

Were we disposed to enter into a minute criticism of Dr. Fletcher's work, (which we are only prevented from doing by the length which this article has already reached, and the ground we have yet to pass over,) we should feel it necessary to point to much that we regard as fallacious, as well as to express our approbation of much that is sound and valuable. On a few topics of importance we shall record our dissent from him, in order that the many into whose hands his work will probably fall may be led to consider how far the weight of his arguments and the ingenuity of his reasoning establish the positions he endeavours to prove. These are sometimes invalidated by the inaccuracy of the facts he has assumed as their foundation: thus, in the comparative classification of plants and animals, which he has introduced into his first chapter, the Cryptogamic tribes are placed opposite to the Zoophytes and Mollusca; the Endogens are regarded as corresponding with Fishes, and the Exogens with the higher Vertebrata; the sub-kingdom Articulata being left without any analogous division in vegetables. Now, if any correspondence exist between the principal divisions of the two kingdoms, we cannot help recognizing that of the Endogens and Articulata as more evident than can be discerned between any other groups; and this analogy was pointed out by Desfontaines soon after his grand discovery of the essential difference in the structure of the Exogens and Endogens. A few pages subsequently we find it stated that no remains of Coniferæ, or other dicotyledonous plants, are found beneath the tertiary strata; although the fact is now quite notorious that nearly the whole of the coal formation is to be regarded as made up of the partially decomposed trunks of the pines and their allies. These inaccuracies, which are among the first that strike us on opening the volume, are, we regret to say, by no means solitary; and it is manifest that, however acute may be the reasoning founded upon fallacious statements, the results cannot be looked on but with suspicion. Whilst, therefore, we express our approbation of such chapters as that on the Nature of Life, where the clearness of the author's mind is peculiarly well displayed in grappling with the abstract conceptions which the subject necessarily involves, we must put our readers on their guard against similar displays of apparently conclusive reasoning built upon unsound premises. Thus, immediately after the vital properties have been proved to be endowments of organized matter, we find the author denying such vital properties to the chyle and blood, forgetting that both these fluids exhibit more or less of an organized structure; their globules (particularly those of the latter,) presenting an arrangement of their particles fully as complex as that of the simpler solid tissues.*

Still more erroneous appears to us the line of argument in the succeeding chapter, on the seat of Irritability. Believing this property to be confined to the ganglionic nerves, the author assumes, as the basis of his demonstration, that this system is universally distributed, not only through all those parts of animals in which it is known to exist, but even in those lowest organisms in which no nervous fibrils can be traced, as well as throughout the vegetable kingdom; his argument for this assumption being simply, that the contrary cannot be proved. Hence he infers that, as irritability and a nervous system universally coexist, the former is a property of the latter; and, returning to the point in the circle from which he set out, he asserts that vegetables possess a nervous system, *because* they manifest irritability. Now, if we are to assume every thing in anatomy and physiology of which the contrary cannot be proved, we must bid adieu to all hope of elevating them to the rank of exact sciences; and we can scarcely imagine a mode of proceeding more adapted than this to destroy what degree of certainty they have already attained.

From these animadversions we pass with pleasure to the chapters on the Stimuli to Irritability, which contain very much that is valuable, arranged in a clear and well-digested form; and here, as in other parts of the work, the amusing character of the notes relieves the scientific dryness of the text; although it is, perhaps, our duty to criticise these also, as occasionally bordering on that grossness of idea which cannot but offend the truly refined mind.

In the section on Sympathy and Passion, or Instinct, many interesting facts are well grouped together; and we fully agree with Dr. F. in considering the actions excited by these stimuli as of an involuntary character, although we differ from him as to the degree in which the mind participates in their performance: to this subject, however, we shall return in our next Number, when it is our intention to review the whole of the recent discussions on sympathetic and excited actions. We are soon obliged, however, to restrain the expression of approbation; for the following section is devoted to the demonstration of the conveyance of Sympathy, Passion, and Instinct by the Respiratory System of Nerves. We doubt not that these nerves form a part of the general system by which the movements thus produced are excited; but the portion of the instinctive motions which they produce is, to our apprehension, a very small one; and we can see no ground whatever for attributing the *organic sympathies* (which Dr. F. has peculiarly well stated in a collec-

* Dr. F. thinks it superfluous to speak of the vegetable fluids in connexion with this question, (II. *a.* p. 42:) we, on the contrary, think that they have an important connexion with it, as no general statement can be correct which will not include all the cases to which it applies. We may refer our readers to the curious observations of Amici on the formative power of the elaborated sap of the vine, (*Ann. des Sci. Nat.* t. xxi.) and to those of Mr. Knight on the Vegetable Fluids. We may also take this opportunity of mentioning a fact to which we do not remember to have seen anything analogous stated elsewhere: a patient, supposed to be labouring under endocarditis, having been bled, the coagulum (formed in a common bleeding-dish,) exhibited at its edges a number of little wart-like processes, exactly resembling in character the softer granulations which are seen on the cardiac valves after death from this disease.

tive form,) to any but the ganglionic system. The universality of the distribution of the respiratory nerves, which is our author's main argument, is a mere assumption, and is only presumed to exist from the connexions between the pneumogastric and the sympathetic: we are here, therefore, again called upon to protest against such an unstable foundation being employed as the basis of a train of reasoning, the ingenuity and connectedness of which might well deceive the unwary into a belief in its solidity.—We shall here take our leave of this work; and in doing so shall again express our regret that its highly-gifted author did not live to bring it to a termination, and that the cultivators of the science of physiology have been deprived of the aid of one so peculiarly fitted, by his extensive information and acuteness of intellect, to promote its interests.

IV. The progress of our review now brings us to a work planned and executed in a very different style, which we regard as peculiarly characteristic of the English School of Physiology; but, before proceeding to a detailed examination into its merits, we shall offer a few remarks on the progress of the science in this country since the time of Haller, and point out what may be considered the principal advantages and disadvantages of the method in which it is usually cultivated in Britain. Although Glisson had, long anteriorly to Haller, propounded in a general form doctrines similar to those which Haller particularly applied to the muscular structure, and which, as we have already shown, his successors extended to other systems, the little influence they had acquired in the country where they originated is shown by the reluctance evinced by the British physiologists to give up the favorite tenet of the then predominant school, that all the vital properties of the tissues are referrible to the influence of the nervous system. The keen discussions carried on by Whytt and Porterfield with Haller and his followers, doubtless, contributed to the advancement of theoretical physiology in this country; although the effect of the writings and instructions of the first-named author is almost as evident in the more recent productions of our speculatists, as is that of the sounder and more philosophical doctrines of Haller on the works of the German school, or at least of that portion which has not been mystified by the abstractions of transcendentalism. There are few English physiologists, even of the present time, who do not in their writings acknowledge, or even defend, the notion of a distinct controlling agency in every living system, although every writer has his favorite designation for this fiction of the imagination. We can imagine nothing more calculated than this doctrine to check the real progress of physiological enquiry; since, to refer every inexplicable process in the living system to the operation of a Vital Principle is about as conclusive a method of saving the trouble of further investigation as that of Molière's candidate, who, to the "Quapropter opium facit dormire?" of the examiner, replies with his "Quia habet vim dormitivam." The influence of the doctrines of Whytt was aided and strengthened by the investigations of Hunter; and fully conceding, as we do, the great merit of the latter in establishing the operation of vital properties in the various processes of the animal economy, both healthy and diseased, it is a source of regret that his name has assisted to keep alive, especially among his friends and pupils, a system which we are convinced he would, if now alive, do his utmost to overthrow.

It has been a necessary consequence of the prevalence of the doctrine of a vital principle, that little pains have been taken by English physiologists to ascertain the elementary vital properties of the tissues and the laws of their operation; a study which, on the continent, has been so fruitful and important in its results. Even the brilliant theory of Brown excited but temporary attention in this country, and was forgotten almost as rapidly as it was brought into notice; whilst, as we have seen, it assisted greatly in the revolution in speculative physiology which was taking place in Germany, by directing attention to the external agencies on which vital actions are as much dependent as on the vital properties of the organism. Still, however, our countrymen have not been idle; and their labours, directed in a different channel, have been eminently successful in the departments chosen for research. The British School of Physiology is essentially a practical one; the object which it has kept steadily in view has been the discovery and establishment of facts; and it has been particularly advantageous for its progress that it has been generally unincumbered with those premature theories and fanciful speculations which have frequently exercised an influence over continental physiologists, of which they were themselves unconscious. Although, as we formerly observed, the number of labourers in this department of medical science has hitherto been small in Britain, compared with that which is to be found amongst the continental faculty, this very circumstance perhaps increases the value of the results obtained by the former; since it is to be supposed that none embrace the pursuit who are not conscious of its importance, and determined to advance its true interests. Moreover, there is in this country a very strong and, we think, a very proper feeling against that indiscriminate employment of experiment which characterizes some of the continental schools; and the objection applies not only to the cruelty, but to the worthlessness of the practice. Hence the results of experiments made with caution, and intended more as a verification of opinions founded upon other grounds, than as a *means* of discovery, are evidently more trustworthy than those of trials made at hazard, with no definite end but the expectation of some striking result. Whilst, therefore, speculative physiology may certainly be regarded as less advanced in Britain than on the continent, we cannot but feel that the progress which the science has made in this country may be considered as strictly inductive, and that the materials which have here been collected are worthy of being placed amongst the most valuable and important which any country has contributed. We cannot forget that it was Hunter who first gave prominent expression to the fact that the truths of physiology are to be sought, not in one or two systems alone, but in every variety of state of each living organism, whether animal or vegetable; and the influence of his life and labours has, we are certain, done far more than counterbalance the injurious effects of some of his doctrines, by the earnestness with which he inculcated and practised that freedom and extent of enquiry against which no erroneous dogmas, however supported by authority or sanctioned by time, can long maintain their ground. The work of Dr. Fletcher affords a notable instance of the baneful influence of preformed theories in warping or distorting facts. Wherever the author has employed his acute powers in reasoning from the known to the unknown, his conclusions are stable and definite; but, as soon as he admits the influence of a favorite theory, his ingenuity is misemployed in torturing the evidence upon which it is to be established.

Notwithstanding, however, the great advantage which has accrued to the English School of Physiology from its attachment to facts and disregard of hypotheses, we cannot but think that there is rather too much caution exercised regarding the admission of some of those generalizations which have been fairly established by observation and experiment. The work of Dr. Roget was the first in which the ascertained laws of development were placed before the British public; and we have heard the author severely criticised by men of some eminence in our profession, even for the very moderate, and we should have thought unobjectionable form in which these doctrines were put forth. That of Dr. Fletcher has taken a still bolder step, yet one fully justified, in our opinion, by the mass of facts he has accumulated in its defence; and we look forwards to the time when the doctrines to which we have alluded shall not only be received upon sufferance, but confirmed and extended by the labours of our countrymen. They certainly stand upon a much surer foundation than the shadowy *Archæus* which has so long held dominion amongst us. We should be sorry to see less eagerness in our schools for the observation and collection of facts; and an exclusive devotion to this object is certainly less practically injurious than a complete reliance upon theory. It is well that there should be in physiological science, as elsewhere, a conservative section, who may restrain the movement party from advancing with unsafe rapidity: and it has always appeared to us a very beautiful adaptation of the varieties in the constitution of the human mind to the external world, that there should be this balancing of tendencies. Nevertheless, we should like to see more regard paid in this country to physiology as a *science*,—that is to say, as an expression of general principles, and not a mere collection of facts.

The favorable reception which the former editions of Mr. Mayo's Outlines have experienced from the medical public might appear to render superfluous the expression of our opinion regarding the general merits of the work, since these have already been pronounced upon by a tribunal to which ours must be regarded as subordinate. We cannot avoid, however, pointing out the features which peculiarly distinguish it from other elementary treatises on the same subject; and it is then our intention to notice the principal improvements which have been made in the present edition, and to advert to those of which we consider it still susceptible: and, if it should appear to our readers that we bestow a disproportionate degree of attention on the last-named topic, we hope it will be believed that, far from wishing to depreciate in this manner the estimation in which these Outlines are deservedly held, we are only anxious that the work should be rendered still more worthy of general regard.

The main purpose of the work is to afford to the student of physiology an outline view of the facts at present recognized in the science; and, keeping this end in sight, its author has in most cases studiously avoided entering on the discussion of disputed questions, and has exercised a due degree of caution in the admission of those generalizations (many of them more specious than solid,) which some of the continental writers on this subject are so fond of putting forward. The facts are in most instances well selected and arranged, and detailed in clear and concise language; so that, without exhibiting the comprehensive learning of Bostock, or the profound talents of Alison, Mr. Mayo has presented to the English student a work free alike from the embarrassing richness of the "System" of the former writer, and from the somewhat abstract refinements which perplex the ordinary reader of the "Outlines" of the latter. Hence we should say that, as an elementary treatise, we regard Mr. Mayo's as the most practically useful; whilst the more advanced student may receive a large increase to his knowledge of the history and facts of his science from Dr. Bostock's work, and of its principles from that of Dr. Alison. When the science of physiology shall have been so far simplified as to render a knowledge of the structure and functions of any particular being a process of simple deduction, then, we feel convinced, the acquirement of its *principles* should be the first object; but we doubt if this can, with most minds, be attempted in its present condition. Having already quoted the opinions of authors of the German School on the most essential doctrine of physiology, that of a Vital Principle, we shall bring into contrast with them a brief extract from Mr. Mayo's first chapter, which contains the clearest statement we have hitherto met with of the true signification of the words *law*, *property*, and *principle*.

"The word *law* serves to express the conditions essential to a change; the word *property* attributes to a substance the power (capability) of producing a change under ascertained conditions; while the word *principle*, characteristic of a less advanced state of science, has been generally employed (as the final letters of the alphabet are used by algebraists,) to denote an unknown element, which, when thus expressed, is more conveniently analyzed. . . . Hitherto the laws of life, or the properties of living matter, have not been determined with precision; and physiologists have shown themselves reluctant to disuse the vague term 'a principle of life,' to which an imaginary and delusive meaning is attached by many, who forget that terms of this nature have no real value, except as generalized expressions of facts." (P. 2.)

With the second chapter, on the Blood, we are sorry to feel called upon to express considerable dissatisfaction. Although much addition has recently been made to it, the author has neglected many observations of superior importance, which ought unquestionably to have been introduced. No notice is taken of M. Lecanu's analysis of the blood, or of his interesting enquiries into the varieties it presents in the proportions of its solid and fluid parts, according to age, sex, temperament, previous depletion, and various constitutional states. The observations of Hewson on the form of the globules are also omitted; and we are the more surprised at the neglect of this most accurate authority, because his statements, though denied by Messrs. Hodgkin and Lister, have been confirmed by MM. Prevost and Dumas, and still more recently by Professor Müller and M. Milne Edwards; the two latter of these observers having arrived at the same conclusions independently of each other. But the greatest omission is that of the view of the constitution of the blood in the living state, which originated, we believe, with Hewson, and which, after steadily gaining ground in this country, has been proved to demonstration by Müller. Although Mr. Mayo has entered pretty fully on the causes which influence the rapidity of the coagulation, he has neglected one remarkable and important fact, that it is most rapid where the proportion of serum to crassamentum is largest, and especially where this arises from previous loss of blood, which bears strongly upon the *quæstio vexata* of the vitality of the blood. Indeed, we think that he has given his opinion rather hastily, when he says that the phenomenon of coagulation may be regarded as the result of the chemical composition of the blood, since it has led him to pass by the very interesting connexion which it has been shown to have with the rigidity of muscles after death, as well as many other facts and arguments which have been advanced in support of the contrary position.

To the chapter on Muscular Action we do not perceive that any addition has been made, nor perhaps is much required; though a notice of Nysten's experiments on the irritability of various muscles after death, as tested by the effects of galvanism, might have been advantageously introduced. The classification of the muscles according to the nature of the stimulus by which they are excited, is, we believe, peculiarly due to Mr. Mayo, and probably leads, more than any other, to a just appreciation of their characters. The primary divisions of voluntary and involuntary muscles are formed by the possession or want of sensibility to the stimulus of mechanical irritation, conveyed through the nerve distributed to each. The strictly involuntary muscles contract only by the application of a stimulus to themselves, and their action is usually unattended with consciousness, and cannot even be controlled by the will. The class of voluntary muscles, on the other hand, contains not only those which respond to mental influence, but those which perform many automatic movements without the aid of that influence, and even in opposition to it. Mr. Mayo appears to have some difficulty in subdividing this class according to the actions of the muscles, since they are all alike called into play by nervous influence; and he does not seem to conceive it possible that such influence can be transmitted without an effort of the will. On this subject we may refer to some observations in our last volume, (p. 421,) which appear to us to set the involuntary action of the reputed voluntary muscles in a clear light: this is not to be confounded, however, with that of the purely involuntary muscles, since the former still depends upon the stimulus of innervation, whilst the latter cannot be excited by it.

In his chapter on the Mechanism of the Circulation, we are sorry that Mr. Mayo has not more fully availed himself of the many valuable results which have been laboriously and carefully attained since the publication of the former edition of his work. Thus, when speaking of the action of the mitral valve, he states that, during the systole, the direction of the chordæ tendineæ is oblique, and not in the axis of the ventricle; so that the two opposite edges of the valve are brought into contact by their tension. Now, the Dublin committee, appointed by the British Association, directed special attention to this point, and came to the conclusion (in which they were partly confirmed by the Report of the London Committee,) that the action of the chordæ tendineæ does not produce closure of the valve, that being effected by the pressure of the refluent blood at the moment of systole; but that it draws the flaps of the valve into the most advantageous position for being thus pressed together.

Another remarkable instance of Mr. Mayo's complete neglect of these valuable Reports occurs soon afterwards. "The first sound of the heart is probably caused by the flapping-to of the mitral valve, and the protracted vibration of the tendinous cords; the second, by the flapping-to of the sigmoidal valves. It was found by Dr. Hope, that the preventing either valve from closing, by wires passed into the heart, put an end to or impaired the sound attributed to the shutting of that valve." In opposition to this statement, we might bring forward the numerous experiments of the London Committee, which appear to prove that the auriculo-ventricular valves are incapable of producing a sound analogous to the flapping-to of the sigmoid valves; and that, if their action be prevented, the first sound is scarcely diminished. With respect to the relative value of these statements, our readers must use their own judgment; but we cannot help being surprised that the results of investigations, so important in their character, and so fairly and ably conducted, should have been neglected in a treatise like the present.

Without stopping to discuss Mr. Mayo's theory of Inflammation, the objections to which have elsewhere been fully stated,* we must remark, that the author has left entirely out of consideration all those phenomena which satisfy us that the circulation of blood in the living system is not altogether dependent upon the impulse of the heart and the contraction of the arteries. It is, perhaps, desirable in an elementary work to avoid entering largely into the discussion of controverted questions; but the leading facts on each side should be fairly stated in such a form as to encourage further enquiry on the part of the student. In this particular instance, too, the enquiry into the nature of the capillary circulation, the causes by which it is maintained, and the influence it undergoes from external objects, may be regarded as a question of the highest practical importance; and we are therefore the more surprised that it has been here overlooked.

Little alteration has been made in the chapter on Pulmonary Circulation, but one of the additions to it is entitled to notice. "It is evident that, when we view the carbonic acid of expiration as directly thrown off from the blood, it must be classed as a secretion. If so, it forms a remarkable instance of a secretion that can be made to take place after death." (P. 64.) With the view expressed in the first sentence we fully coincide. The structure of the lungs, when regarded in its general conditions, is precisely analogous to that of the acknowledged excretory organs; nor can any reason be assigned why the exhalation of carbonic acid from the lungs should not be placed on the same footing with the transpiration of aqueous fluid from the skin. If regarded in the light of a secretion, we certainly have it in our power to trace much more clearly than in any other case the chemical changes essentially concerned in it; but does not the rapid advance of organic chemistry encourage the hope that we shall be able, ere long, to obtain as full comprehension of other processes of the animal economy as we now possess of the function of respiration? In considering the organic changes which take place after systemic, or (as Dr. Prichard suggests) *somatic* death, we must bear in mind that none of these can be much prolonged unless the means are supplied

* Supplement to Alison's Physiology, pp. 19, 20.

by the continuance of the circulation, which is the common bond between them all. Some also require the aid of the organs of animal life; but it may, we think, be stated as a general fact, that even when sensation and volition have ceased their manifestations, and nervous agency is totally extinct, each one of the organic functions may continue as long as vital properties remain in the tissues which perform it, and the necessary means are supplied. Thus, the process of respiration may undoubtedly be performed after death, without artificial aid; for the heat frequently returns to the body, and the livid hue on the faces of those who have died from asphyxia often gives place to a vermilion tint: there can be little doubt, therefore, that a similar change is taking place in the capillaries of the lungs, if oxygen be in contact with them. But, in order that these processes may *continue* to be performed, there must not only be a movement of blood in the vessels, but also a renewal of oxygen in the lungs; and hence the artificial assistance sometimes given by the inflation and compression of the thorax cannot be regarded as *producing* the changes which essentially constitute the function of respiration, but only as enabling them to be prolonged.

Amongst other omissions that we feel called upon to notice is one which is calculated to give to the student a very erroneous view of one of the most important practical questions in physiology: we mean the cause of death by asphyxia. Mr. Mayo repeats in the present volume the statement which we find in the former edition, that life is destroyed by the continued suspension of the energy of the brain, occasioned by the circulation of venous blood; and that the heart's actions are checked by the loss of muscular irritability, from the same cause. Had the opinions of Drs. Williams and Kay been mere hypotheses, they might fairly have been excluded from a work which professes to give a view of that only which may be regarded as ascertained in the science; but, as these opinions are supported by numerous well-devised and carefully conducted experiments, which no one has attempted to invalidate, they should, we think, take the rank to which, as legitimate inductions, they are entitled. That the circulation of venous blood in the brain does not immediately produce cessation of vital actions, has been fully proved by Dr. Kay; and that some other cause than the want of the stimulus of arterial blood is concerned in extinguishing the irritability of the heart, appears probable from the experiments of the same author, and also by those of Dr. J. Reid,* who has shown that, when the action of the right ventricle ceases in cases of venous congestion, it may often be restored by such a slight abstraction of blood from the cavity itself or the veins leading to it, as will relax the over-distended fibre; a fact, the practical importance of which it is needless for us to point out. And, finally, we regard it as almost positively established that the chemical changes for which the pulmonary circulation is adapted, are essential to the continuance of the flow of blood through the capillaries of the lungs; and that the cessation of the respiratory movements only acts secondarily upon the circulation, by impeding these changes. We do not call upon Mr. Mayo, however, to exchange his opinion for ours; but simply to give that attention to the evidence on the subject which it certainly merits.

* Edinburgh Med. and Surg. Journal, vol. xlv. p. 387.

The sections devoted to Secretion and Animal Heat claim but little remark from us, since they present a very fair outline of the present state of our knowledge on these topics. We may remark, however, that many facts, besides the *one* mentioned by Mr. M., confirm the idea that the ingredients of the excretions pre-exist in the blood; and that there is much evidence, not adduced by the author, to prove that animal heat is principally maintained by the chemical changes constantly taking place in the tissues. We are ourselves inclined to the belief that the influence of the nervous system upon the temperature of the body is only secondarily exercised by the control which it certainly possesses over these molecular changes; and this belief derives confirmation from the remarkable facts with which vegetable physiology supplies us, and to which we have alluded in a former article. We cannot leave the subject of secretion, however, without expressing our great satisfaction with the very clear and concise analysis which Mr. Mayo has given of Mr. Kiernan's discoveries in the anatomy of the liver.

On the chapter on the Functions of the Stomach we have only to observe, that no allusion has been made to Brâchet's experiments on the influence of the par vagum on chymification. We do not refer to them as having solved this difficult problem, but as offering one out of many possible explanations; and the circumstance of the Monthyon prize having been adjudged to their author may be regarded as entitling them to some notice. If Brâchet's experiments be correct, there can be little doubt that the function of the par vagum is, on the one hand, to convey to the sensorium the impression that produces desire of food; and, on the other, to produce those motions of the parietes of the stomach which are necessary to the first process of digestion. It is fair to state, however, that some of the experiments we have ourselves witnessed do not altogether confirm those of Brâchet; but any one who is acquainted with the complicated distribution of this nerve must perceive the difficulty which is created by its numerous anastomoses, in arriving at any certain results. If the nerve be divided between the superior and inferior laryngeal branches, their connexion will, to a certain extent, keep up the nervous current; and, if the trunks only are divided where they accompany the œsophagus, the plexus formed upon that tube is quite sufficient for the temporary supply of nervous agency to the functions of the stomach.

We are sorry to be obliged to continue our notice of omissions by pointing out one that relates to a question in physiological anatomy, which is still exciting the highest interest; namely, the origin of the lacteals and lymphatics, and the nature of their communication with the veins. On the former point, Mr. Mayo cites the old observation of Cruikshank, and omits to mention that almost every modern physiologist has now abandoned the idea that the absorbents commence by open mouths on the villous coat of the intestines; even Magendie, who long retained this opinion, having now become convinced of the contrary. The question of the communication of the circulating and absorbent systems is, we admit, still open to controversy. Whatever weight be attached to the experiments of Fohmann and Lippi on the affirmative side, at least equal credit is due to those of Rossi and Panizza on the negative; and these should scarcely have been omitted by Mr. Mayo.

We are glad to be able again to exchange the tone of censure for that of commendation in regard to the addition, which next presents itself, of an excellent outline of M. Breschet's observations on the structure of the skin; and still more respecting the chapter on the Nervous System, which now comprehends an extended view of its anatomy, completed by the introduction of the latest discoveries, with much that is interesting and useful regarding its functions. It will be seen, from our former statements, that we differ widely from Mr. Mayo respecting the nature of instinctive actions: he considers them as the result of unavoidable impulses acting through volition; whilst we regard them as independent of volition, and produced by the direct influence of external impressions operating through the cerebro-spinal axis, and the circle of nerves proceeding from it. Experiment and observation alike prove that the hemispheric ganglia are not necessary to the performance of the instinctive actions immediately concerned in the maintenance of life; and it is difficult to conceive that sensation and volition can reside in the spinal cord without any other mental faculties. Whatever view we take of this question, however, we fully agree with Mr. Mayo in the belief that almost all animals enjoy, in a greater or less degree, the possession of various mental faculties, analogous to those of man, and superadded to their instinctive powers. These mental faculties appear to us to give the capability of education; and their extent in different tribes of animals would seem to bear some general relation with the development of the hemispheric ganglia, or the parts corresponding to them in the Invertebrata. The instincts themselves can scarcely be modified, except by the controlling power of the will, or by some alteration in the organization of the being, adapting it to a change of circumstances; and this modification is transmissible to the offspring, as Mr. Lyell has beautifully pointed out, only when it is connected with the maintenance of the existence of the animal in its new situation. We could have wished to enlarge more upon this portion of Mr. Mayo's work, unquestionably the most valuable; but our limits compel us to confine ourselves to his pathological doctrines (now introduced for the first time in these "Outlines,") on which, as they have not been previously noticed in our Review, we shall offer a few observations. These we shall introduce by reminding our readers of the anatomy of the medulla oblongata, in which, as is well known, some important discoveries have recently been made.

According to Mr. Mayo's description, "The greater part of each half of the medulla oblongata,—that is to say, all but the corpus restiforme and the anterior pyramid,—is continuous with a tract, which ascends behind the pons Varolii to the cerebral hemisphere of the same side; and therefore places that cerebral hemisphere in mechanical communication with the nerves of sense and motion of the same side of the body." Mr. Solly, however, seems to regard this tract as commencing with the *sensory* column only of the spinal marrow, and as terminating in the thalamus of the same side.* With regard to the corpora restiformia, Mr. Mayo, after alluding to Mr. Solly's discovery of their communication with the anterior column, concludes that, by their means, "each hemisphere of the cerebellum is placed in mechanical communication

* Solly on the Brain, p. 226.

with the origins of the nerves, both motor and sentient, of its own side of the frame." If Mr. Solly's idea, that part of the cerebellic fibres proceeding from the motor column enter into its decussation,* prove correct, it is evident that each hemisphere of the cerebellum will then be connected with both sides of the spinal cord. Mr. Mayo then proceeds to describe the decussation of the fibres of the corpora pyramidalia. He first shows that "the anterior pyramid of one side is placed in mechanical relation with the corresponding hemisphere of the brain;" and then states that "a thin layer of the outer filaments seems to spread, some to the corpus restiforme, some to the anterior surface of the same half of the spinal marrow, but by far the greater part bend over to the opposite half of the spinal marrow, dipping towards its centre, into which they plunge in such a manner as to place themselves in mechanical relation with the anterior and posterior parts of the opposite half of the cord."

Here we again find Mr. Solly differing from Mr. Mayo; the former author regarding the corpora pyramidalia as exclusively belonging to the motor tract, which commences in the corpus striatum, and terminates in the anterior column of the spinal cord. We mention these varieties in the opinions of anatomists who have paid great attention to the subject, as proving that we are not yet in a condition to draw any very definite conclusions from pathological phenomena with regard to the influence of cerebral lesions on the functions of the nervous system; a fact which is acknowledged by the most eminent authorities. Mr. Mayo has, however, advanced a theory on the subject (explained at greater length in his "Outlines of Pathology,") which he considers as giving a satisfactory explanation of the manner in which the decussation of the anterior pyramids contributes to the production of paralysis on the side of the body opposite to that in which the cerebral lesion exists.

"When a cerebral lesion produces palsy, it cannot be supposed to act by interrupting the customary supply of cerebral power to the spinal marrow: for, if it did, the removal of one hemisphere of the brain, or its lateral compression in an animal (from which a large part of the cranium has been removed,) should produce hemiplegia; and an acephalous infant, or an animal with the cerebrum and cerebellum removed, should be completely palsied; which is contrary to fact. Another consequence of this mode of influence, if it existed, would be, that, in palsy strokes, the parts the most remote from the brain would have their supply of nervous energy soonest cut off, which again is contrary to fact; inasmuch as the arm is, in almost every case, struck first, and more severely than the leg, and, when recovery takes place, is slowest in being restored." (P. 222.)

We must say that Mr. M.'s reasoning appears to us somewhat inconclusive. We all know that there are many motions to which the influence of the brain is not essential; these being performed alike in the state of apoplexy, in which volition is entirely suspended or destroyed, in acephalous infants, and in animals from which the brain has been removed. But these movements require only the existence of the spinal cord, and the completeness of the circle of sentient and motor nerves connecting it with the part. Hence there is no more reason why one side of the body should be paralysed by the removal of one hemisphere, than that the whole should be similarly affected by the removal of the entire brain. The fact mentioned in the last sentence of the preceding quotation is

* Op. cit. p. 155.

indisputably correct, but we do not see that it warrants the inference drawn from it. Mr. Mayo probably agrees with us in believing that the motive influence affecting each portion of the body originates in a distinct part of the cerebral mass, although pathologists have hitherto failed in distinctly tracing the connexion: if this be the case, it is very easy to understand that the portion from which the voluntary motion of the upper extremity originates may be more liable to hemorrhage and other lesions than that connected with the lower; and that, being usually more severely affected, it shall be slowest in recovery. It is highly probable, too, that the superior extremity has more extensive connexions with the brain than the inferior; since its actions are more frequently associated with mental operations, both in the needful occupations of barbarian industry and in the finer arts and accomplishments of civilized life. But Mr. M. takes a very different view, and infers, from the facts already adduced, that the cause of the paralysis is "an actively depressing influence propagated from the diseased brain to the nuclei of the segments of the medulla oblongata and spinal cord."* We have already endeavoured to show that there is no necessity for such an assumption, and we shall now point out what appears to us its inconsistency with known facts. If such an "actively depressing influence" be the cause of the paralysis, it is difficult to conceive how it can be propagated to the lower part of the spinal cord without involving the upper, unless that distinctness in the connexions of the nerves with the cerebellum be admitted, which we have shown to render unnecessary Mr. M.'s conclusions. The author admits, as an objection to his theory, that, in hemiplegia from cerebral disease, the leg is sometimes affected alone or in a greater degree than the arm; and allows that, if this occurrence were frequent, it would invalidate his hypothesis. We cannot see why a single decided case should not have the same weight as a hundred, when directly opposed to an hypothesis; but, in the present instance, Mr. Mayo has a mode of escape which would probably not suggest itself to many of our readers: "Occurring as it does very rarely, it admits of many explanations, one of which is the following. Suppose the spinal cord to be predisposed to paraplegia, then the hemiplegic shock supervening and being slight, the phenomenon to be accounted for would naturally ensue." Moreover, it seems to us that the depressing influence ought to annihilate all capability of producing motion where the paralysis is complete,—that is to say, where the power of the will is entirely destroyed; but we have sufficient evidence that this is not the case in the motions which may be excited by internal and external stimuli, in apoplectic and hemiplegic patients. The impulse, according to Mr. Mayo, is received by the spinal cord *en masse*, as it were, and the extent of its propagation is determined altogether by distance. A shock is given to the nervous fluid, and the undulatory

* This idea is not so novel as Mr. Mayo appears to suppose. We extract the following passage from an article on the Physiology of the Brain, contributed by Dr. Gordon to the Edinburgh Review: "The insensibility which follows a division of a nerve, or of the spinal cord, or a destruction of the brain, admits of an equally probable explanation, on the supposition, either that the brain is constantly supplying something to the different parts of the body which enables them to feel, or simply that some injurious effect is propagated downwards along the nerves from the parts which are injured above." —Vol. xxiv. p. 452.

disturbance, though violent in the nearer parts, becomes fainter and fainter till its waves are lost in the more distant. The spinal cord is treated as if it had no distinction of parts; no relation but that of proximity or remoteness to the nerves connected with it. But, if Mr. M. had said that the shock was communicated through the spinal cord in tracks corresponding to the nerves affected, we should not have called his statement in question; for it cannot be doubted that lesions of the brain frequently exert something more than a privative influence,—as in the circumscribed spasmodic and convulsive affections,—whether we call it shock, irritation, sympathy, disturbing or depressing influence, or any term expressing a relation of cause and effect between the central mischief and the peripheral disorder.

We cannot regard Mr. M. as fortunate in his attempt to explain the fact that, in hemiplegia, the face is paralyzed on the same side with the body, although there has been a decussation in the spinal column between the origins of their nerves. This is certainly an occurrence not to have been anticipated, and of a very puzzling character: it does not appear to us more extraordinary, however, than the fact to which Mr. Mayo has not thought proper to allude, that hemiplegia undoubtedly results, in many cases, from cerebral lesion of the same side;* nor do we think that Mr. M.'s theory gives us any assistance. But our readers shall form their own opinion of the value of his explanation. After pointing out that the origin of the portio dura of the seventh pair is above the decussation of the pyramids and just below the pons Varolii, he continues, "Is it surprising that the palsying shock, falling at a point so near this, and in continuity with it by reflected extension of nervous fibrils, should propagate its depressing influence upwards, and paralyze the seventh, the eighth, the ninth, the fifth nerves, one or all?"

We confess we do not understand the drift of this argument. The motor and sensory columns of the cerebro-spinal axis are usually regarded as passing through the pons varolii, (which is formed by the expansion around them of the crura cerebelli,) then proceeding under the designation of the crura cerebri to their termination in the corpora striata and thalami nervorum opticorum. One would naturally suppose, therefore, that a palsy shock originating in the cerebrum would be transmitted downwards along this line, paralyzing in its course the facial nerves arising from it, and would then cross with the decussation of the columns to the opposite side. But Mr. Mayo seems to suppose that the palsy shock is communicated to the cerebro-spinal axis about the origin of the seventh nerve, and then proceeds upwards by "reflected extension" to the facial, and downwards to the spinal nerves. Whence he supposes the shock to originate, he has not clearly expressed, and we cannot therefore be answerable for our want of comprehension. "But how is the fact to be accounted for," he continues, "that lesion of one hemisphere of the cerebellum produces palsy of the opposite side?" Here we encounter the same variety of phenomena as results from lesion of the cerebrum, and to which Mr. M. has not alluded. Although we should expect that as the communication of the cerebellum with the spinal cord is direct, the paralysis should be direct also, it is almost always on the side oppo-

* Andral, Cours de Pathologie interne. Tom. iii. pp. 75-79.

site to that of the lesion; but that it is not universally so, is stated by Andral* and other pathologists. Mr. Mayo's explanation of the crossing of the palsy stroke, does not appear to us more felicitous than his previous attempts at elucidating this most intricate subject.

"A most complex proximity and intertexture exists between the transverse fibres of the pons varolii and those of the anterior pyramid in their progress through it; a closeness of interlacing nearly resembling that in the spinal cord at the mixture of the filaments of the pyramid with it. Is it too bold an hypothesis to suppose that where there is lesion of the cerebellum, the shock which it originates is capable of affecting through that closeness of interlacing the fibres of the pyramid of the same side, which being thus struck, carrying the palsying influence downwards to the point of decussation?"

On this we have only to remark, that Mr. M. does not assert that there is any continuity of the filaments of the pyramids and crura cerebelli, but merely an interlacement; and if simple contiguity is sufficient for the propagation of a palsy stroke, we may bid adieu to all attempts to throw light by anatomical research on the laws of paralytic diseases. With every respect, therefore, for Mr. M.'s ingenuity and pathological knowledge, we cannot see reason for adopting his theory, since it does not seem to us to assist in the comprehension of the complex mass of phenomena which are constantly being presented to our notice. We think that our anatomical knowledge must be much extended and refined before we can pretend to make it a ground of inference; and that, especially, we must gain much additional information on the functions of the cerebrum and cerebellum, and on the minutiæ of the connexions of these organs with the spinal column, and the origins of the nerves. And, in the mean time, we are not ashamed to confess our inability to account for phenomena, which the highest authorities in pathological science have deemed it impossible to explain.

We must now quit this topic, and pass on to the chapter on the External Senses, into which a very important and interesting discussion on the nerves of taste has been introduced. Mr. Mayo still retains, in opposition to Panizza, the opinion that the glosso-pharyngeal nerve is not that which ministers to this sense; but that it principally depends on the third branch of the fifth pair. The arguments drawn from the experiments of Panizza are certainly much weakened, if not entirely set aside by Mr. M.'s counter-statements; and he is, in our apprehension, the most correct in his view of the especial seat of the impression. It is well known that Panizza imagines the sense of taste to be most acute where the filaments of the glosso-pharyngeal nerve are most abundant, viz. towards the base of the tongue. Mr. Mayo, on the other hand, maintains that the sense is most acute at the tip and edges of the tongue, a fact we should suppose conformable to general experience; and hence he infers, that as the branches of the glosso-pharyngeal do not reach the tip of the tongue, the fifth must be the nerve which conveys the impression from those parts. From various experiments, he concludes that the former nerve does not act upon the muscles of the tongue; and from some pathological observations, he thinks it probable that it conveys the vague impressions of contact and nausea which are produced on touching the base of the tongue. The question is one which is fully open to fur-

* Op. cit., pp. 84, 85.

ther investigation, especially since the experiments of Mr. Broughton and Dr. M. Hall, communicated to the meeting of the British Association at Bristol, completely accord with those of Panizza, (several facts drawn from comparative anatomy seeming to afford confirmation to their views;) whilst those of Dr. J. Reid laid before the last assembly of the same body, (in which our personal knowledge of that gentleman's skill and accuracy leads us to place great confidence,) go to prove that it is a nerve of common sensation, and that it is by the impressions conveyed by it that the motor nerves of the muscles of deglutition, (principally the pharyngeal branches of the par vagum,) are called into action.

In the other sections of the same chapter which have been added in the present edition, the reader will find much that is interesting on various modifications of common sensation. The sense of effort must certainly be admitted as one of these; but we can scarcely say the same of the sense of motion, which seems but a variety of the former, occasioned by the temporary resisting action of the muscles when the motion which the body is sustaining is altered either in rapidity or direction. In the section devoted to some of the uses of sensation, which is well calculated to bring the student's knowledge into exercise, we find one passage which strikes us as somewhat infelicitous.

"The idea of *outness*, on which our belief in an external world, in place of a world of dreams, is mainly founded, is not excited by every sense. Smelling, tasting, hearing, do not appear to me to excite it; nor motion necessarily: but it is irresistibly conveyed to our minds by sensations of touch, resistance, and vision. In the last instance alone the fact may appear questionable; yet it is sufficiently proved by the remarkable expression of Cheselden's patient, who, when first restored to sight, drew back, thinking that the objects which he saw touched his eyes." (P. 323.)

Now without stopping to criticise the word *outness*, we may ask whether the sensations of touch and vision are not as often and as fully experienced during sleep as those of smell, taste, and hearing; and how, therefore, our belief in the reality of an external world can depend peculiarly upon them? Cheselden's patient had probably not been disturbed with any metaphysical doubts on this knotty question; and nothing could be more natural than that the impressions derived through his new sense, should be at first associated with those by which most of his knowledge of external objects had been previously acquired. An interesting case occurs to us which we shall mention, although not altogether in point. Some years ago, we saw the operation for congenital cataract performed upon an intelligent boy of between four and five years old. For some time after the pupil had completely cleared, he found his way about his father's house and other places to which he had previously been accustomed, more by touch than by sight; but in a new situation the latter sense was of material assistance to him.

Passing by the division of the work appropriated to Voluntary Motion, which contains few additions of importance, we proceed to that which embraces some of the most interesting questions in physiology, namely, those connected with the function of reproduction. Few can be ignorant of the nature of the beautiful researches in embryology, which have recently occupied the attention of our continental brethren, but which, with a very few honorable exceptions, have received no advancement in this country. The tendency of these researches has not only been the collection of facts, but the establishment of certain general laws derived by induction from them, and opening to us views of the highest interest. In the former edition of Mr. Mayo's work, we were much pleased to observe the attention which he had evidently given to the subject, and the lucid manner in which the materials drawn from numerous sources had been digested and arranged. These excellencies, which it possesses in common with several other parts of the treatise, are not diminished in the chapter as it stands at present, since it has undergone but little alteration; but the progress of this department of science, in the interval, fairly required from Mr. Mayo the recasting of the whole of the section appropriated to it. The extent of the subject forbids us from entering into those details which we have thought it desirable to introduce on other topics; we may, however, specify some of the deficiencies which most forcibly strike us.

It appears to be now established as a general fact, that the fundamental or essential structure of the ovum is the same, not only among vertebrata, but in most, probably all, of the classes of the invertebrata; and that the peculiarities which afterwards characterize the different tribes arise only in the progress of development. The order in which they arise necessarily results from the passage of a more general type into a more special one; thus, in the development of any ovum, the characters of the sub-kingdom are first superadded to those of the animal; the characters of the class appear after those of the sub-kingdom; and so with those of the order, family, genus, species, and sex. The same law of development, that of a special form being elaborated out of one more general, holds good with regard to the evolution of each individual organ; and as this law may also be easily shown to govern the gradual development of each system in ascending the animal scale, it follows that certain transitory resemblances will be presented by each system of the higher animals during its progressive evolution, with the permanent forms of the same system in the inferior classes. Now this being one of the highest generalizations which we can expect to attain on this subject, and being in our opinion firmly established, it would have given us much pleasure had we found that Mr. Mayo had arranged his abundant materials in such a manner as to illustrate and confirm it. With regard to the first point, the essential correspondence in the structure of the ova in all classes of animals, he is altogether silent; he does not even mention the names of Purkinje, Valentin, Wagner, Coste, and Delpech, to whom (especially to the first three) we are indebted for this beautiful generalization; and we find also that he retains the description of the ovulum of mammalia given by Von Bär, notwithstanding that the researches of Purkinje, Bernhardt, and Valentin, have distinctly proved that the ovulum instead of being analogous to the germinal vesicle of birds, as Von Bär supposed, really corresponds with their whole ovum, and contains a yolk and germinal vesicle.

We must close these remarks by noticing an interesting observation recently made by Mr. Mayo on the character of the globules in pus.

"I have ascertained that the particles of pus bear the following relation to those of the blood. They are exactly of the same shape; that is to say, they resemble in form silkworms' eggs, being circular flattened discs with rounded edges, having a shallow circular depression on each flat surface. They were previously known to be circular and to be larger than blood-particles. Measuring them, I found their diameter to be $\frac{1}{2000}$ of an inch; while, in the same micrometer, the particles of blood appear to be $\frac{1}{3000}$ of an inch in diameter. Particles of pus are therefore, without doubt, particles of blood slightly changed." (P. 475.)

In the foregoing criticisms, it has been our principal object to direct the attention of our readers to the chief novelties which either are or ought to have been introduced into the present edition of Mr. Mayo's work. We are far from wishing to diminish the estimation in which it is universally held; and we should have taken more notice of the many excellencies by which it is distinguished, did we not suppose them to be already well known to our readers. Indeed, it wants but to be continued with the same zeal and industry with which it was first compiled, to be rendered one of the best elementary works on Physiology, not only in our own but in any language.

V. The last work on our list is, we believe, the only systematic treatise on Physiology that has in recent times appeared on the other side of the Atlantic; and we hail its publication as the harbinger of yet more important contributions to this branch of medical literature from the same quarter. Dr. Dunglison has aimed at nothing more than the selection and arrangement of the facts dispersed through the works of the numerous authors who have written on various branches of physiological science; and he appears to have performed his task with much judgment, his production being distinguished by the same lucidity of thought and clearness of expression, which characterized those which we have formerly noticed with approbation. The student who consults it must not expect, therefore, to meet with comprehensive views or ingenious theories; but he will find it a storehouse of details carefully selected, and judiciously arranged. In this respect it may be considered intermediate between the works of Dr. Bostock and Mr. Mayo; being free from the perplexing profuseness of the one, and having the advantage over the occasional meagreness of the other. The judgment of the author is frequently shown to considerable advantage, in the concise summaries which he appends to the conflicting statements of different observers; the want of which is the principal fault we have to find with the learned system of Dr. Bostock. Were we inclined to criticise the plan of Dr. Dunglison's treatise, we should suggest that the anatomical descriptions which are to be found in every elementary work on the structure of the human body, are somewhat superfluous and add unnecessarily to the bulk of the volumes; although we must admit, that no work on physiology can be complete or even comprehensible which does not embrace many structural details not usually dwelt on with sufficient minuteness in anatomical works, as well as much illustration drawn from comparative anatomy; and for a treatise like Dr. D.'s, which seems intended to be accessible to the unprofessional reader, there is, perhaps, an advantage in combining a general description of the arrangement of the organs with the account of their functional manifestations. If Dr. D. had substituted for these descriptions, however, more copious references to the organization of the other tribes of animals, we cannot but think that he might have rendered his work more generally useful to all classes of his readers. In a treatise on human physiology which considers man merely as an *animal,* it is perhaps correct to commence with the description of his most purely animal organs and functions, which is the course adopted in the present work; but when man is regarded as having an *organic* life distinct from his animal life, and supplying the latter with the conditions of its existence, we cannot but think that it is the most philosophical, as well as the most convenient arrangement, to begin with the consideration of those functions which are common to all classes of organized beings. This, however, is a matter of opinion; and almost every systematic writer has shewn that he differs in this matter from his predecessors.

It is obvious, from what we have stated of the plan of Dr. Dunglison's treatise, that the details of its execution afford little food for criticism; and we shall therefore content ourselves with transferring to our pages some of the few novelties most likely to be interesting to the English reader, and making a few extracts which may convey an idea of the general style of the work. The following remarkable case of the effect of the inhalation of nitrous oxide gas is related by Professor Silliman.

"The subject of the case was a man of mature age, and of grave and respectable character. For nearly two years previous to his taking the gas, his health had been very delicate, and his mind frequently gloomy and depressed. This was particularly the case for a day preceding the inhalation, and his general health was such, that he was obliged to almost wholly discontinue his studies, and was about to invoke medical aid. In this state of bodily and mental debility, he inspired about three quarts of nitrous oxide. The consequences were, an astonishing invigoration of the whole system, and the most exquisite perceptions of delight. These were manifested by an uncommon disposition for pleasantry and mirth, and by extraordinary muscular power. The effects of the gas were felt undiminished for at least thirty hours, and, in a greater or less degree, for more than a week. But the most remarkable effect was on the organs of taste. Before taking the gas, he had no peculiar choice of food, but after this, he manifested a taste for sweets only, and for several days ate nothing but sweet cake. This singular taste was, indeed, carried to such an excess, that he used sugar and molasses, not only upon his bread and butter, and lighter food, but upon his meat and vegetables. This he continued to do until the time when Professor Silliman wrote, eight weeks after the inhalation, when he was still found pouring molasses over beef, fish, poultry, potatoes, cabbage, or whatever animal or vegetable food was placed before him. His health and spirits were good, and he attributed the restoration of his strength and mental energy to the influence of the nitrous oxide." (P. 119.)

In the chapter on the physiology of the circulation, we find the following interesting fact regarding the irritability of the heart, which is recorded by Dr. Mitchell of Philadelphia.

"In 1823, being engaged in dissecting a sturgeon—*Acipenser brevirostrum?* its heart was taken out and laid on the ground, and, after a time, having ceased to beat, was inflated with the breath, for the purpose of drying it. Hung up in this state, it began again to move, and continued for ten hours to pulsate regularly, though more and more slowly; and, when last observed in motion, the auricles had become so dry as to rustle when they contracted and dilated. He subsequently repeated the experiment with the heart of a *Testudo serpentaria* or *snapper*, and found it to beat well under the influence of oxygen, hydrogen, carbonic acid, and nitrogen, thrown into it in succession. Water also stimulated it, perhaps more strongly, but made its substance look pale and hydropic, and, in one minute, destroyed action beyond recovery." (P. 148.)

We do not recollect to have seen any case recorded of a similar nature to that contained in the following extract, which we transfer to our pages as being alike interesting to the physiologist and pathologist.

"3. The *excretory ducts,* or *biliary ducts.* These are presumed to arise from acini, communicating, according to some, with the extremities of the vena portæ; according to others, with the radicles of the hepatic artery; whilst others have considered, that the radicles of the hepatic ducts have blind extremities, and that the capillary blood-vessels, which secrete the bile, ramify on them. This last arrangement of the biliary apparatus in the liver was well shown in an interesting pathological case, which fell under the care of Professor Hall, in the Baltimore Infirmary, during the last spring, (1835) and which was examined after death, by Professor Geddings, in the author's presence. The particulars of the case are detailed in the 9th No. of the 'North American Archives of Medical and Surgical Science,' with some interesting remarks by Professor Geddings. In this case, in consequence of cancerous matter obstructing the ductus communis choledochus, the whole excretory apparatus of the liver was enormously distended; the common duct was dilated to the size of the middle finger: at the point where the two branches that form the hepatic duct emerge from the gland, they were large enough to receive the tip of the middle finger; and as they were proportionately dilated to their radicles, in the intimate tissue of the liver, their termination in a blind extremity was clearly exhibited. These blind extremities were closely clustered together, and the ducts, proceeding from them, were seen to converge, and to terminate in the main trunk for the corresponding lobe." (P. 252.)

For the same reason we quote the following case, although we doubt whether the details warrant the inference expressed in the introduction.

"The following case, with which the author has been obligingly favoured by his friend, Dr. Wright, has an instructive bearing upon the subject [nutrition of the fœtus.] The condition of the placenta was such as to lead that intelligent observer to conclude, that any circulation between the mother and the fœtus, through the placenta, was impracticable.

"On the 6th of December, 1833, I was requested to visit Mrs. T—, a young woman of large form, good constitution, and generally excellent health. She had been married about fifteen months, and I was now called to attend her first labour. She had felt occasional labour-pains through the day, and was delivered of a fine, vigorous, female infant, in about four hours from the time of my call. The labour was, in all respects, natural, and as easy as is common, or consistent, with a first parturition. After the birth of the child, an hour, perhaps, was passed in waiting for secondary pains to effect the expulsion or favour the removal of the placenta, but no movement of this kind having then occurred, a gentle examination was made to ascertain whether that body might be easily and properly taken away. The vagina contained nothing more than the funis, the outlet of the uterus was open, soft, and extensible. The cord was gently followed into the uterine cavity, and the cake found near its fundus, retaining a close connexion with the uterus. The placental mass was large and firm, presenting to the touch a peculiar feeling, as of a dense sponge, full of coarse, granular, or gravelly particles. Deeming it now proper to relieve the patient fully, a cautious effort was made to detach the placenta from the uterus, in order to its manual extraction. In pursuing this design, it was found, that the adhering surface of the former consisted of a uniform calcareous lamina, or plate, rough to the finger, and exciting such a sensation or feeling as would be caused by a sheet of coarse sand-paper. When the mass was detached, and brought away, the laminar surface just referred to was found to be a calcareous plate, uniformly covering the whole of the attached portion of the cake, the entire surface of the utero-placental connexion. The calcareous matter, thus distributed, was thin and readily friable; but, as before remarked, it appeared to constitute a uniform superficial covering. The correspondent uterine surface, the part from which the placenta had been separated, felt rough, but comparatively soft, imparting nothing distinctly of the calcareous or gritty feel. Out of the body, the placenta felt heavy, and eminently rough throughout. When compressed, or rubbed together, the large amount of nodular or granular matter, dispersed through its substance, was not only manifest to the touch, but a very audible crepitation or grating sound could be thus elicited from any and every part of the mass.

"In this uncommon instance of placental degeneracy, both the mother and child were perfectly healthy and well. The latter, indeed, was remarkable for its fine size, perfect nutrition, and vigour. From the condition of the cake, and the character of its adhesion to the uterus, I apprehended a more than ordinary liability to secondary affection, in the form of puerperal fever; and, whether influenced or not, by the circumstances detailed, secondary fever did ensue on the third day from delivery, attended by the usual signs of puerperal hysteritis, which affection, however, was happily subdued by general and topical bleeding, calomel, &c." (*Note*, p. 409.)

In this very remarkable case, it is certainly a matter of surprise that the fœtus should have attained its healthy size and vigour, which is contrary to the usual result of placental diseases; but it is to be regretted, that Dr. Wright did not ascertain the permeability of the vessels by injection, as the deposit (which, we believe, has been equalled in extent in no recorded instance,*) might have been confined to their interspaces without materially obstructing them.

In closing our notice of Dr. Dunglison's work, we would suggest to its learned author one addition which would much enhance its value; viz. that of references to the authorities from which the facts are derived, especially those which are not usually consulted by the student. We deem it the duty of every one who has traversed such an extensive labyrinth as that which our author has so diligently explored, to leave a sufficient number of direction-posts for the guidance of those that may come after, especially where those hidden and devious paths are to be indicated which might escape the notice of an ordinary treatise.

---

### ART. VII.

* It is to be lamented that Dr. Simpson's valuable monograph on Diseases of the Placenta, (Ed. Med. and Surg. Journ., vol. xlv.) has not yet been completed. Few subjects connected with fœtal development are so deserving of study as the influence of morbid states of the placenta.

